# Untargeted metabolomics reveal pathways associated with neuroprotective effect of oxyresveratrol in SH-SY5Y cells

**DOI:** 10.1038/s41598-023-47558-y

**Published:** 2023-11-21

**Authors:** Nureesun Mahamud, Phanit Songvut, Chawanphat Muangnoi, Ratchanee Rodsiri, Winai Dahlan, Rossarin Tansawat

**Affiliations:** 1 Department of Food and Pharmaceutical Chemistry, Faculty of Pharmaceutical Sciences, Chulalongkorn University, Bangkok, Thailand; 2 The Halal Science Center, Chulalongkorn University, Bangkok, Thailand; 3 Laboratory of Pharmacology, Chulabhorn Research Institute, Bangkok, Thailand; 4 Cell and Animal Model Unit, Institute of Nutrition, Mahidol University, Nakhon Pathom, Thailand; 5 Department of Pharmacology and Physiology, Faculty of Pharmaceutical Sciences, Chulalongkorn University, Bangkok, Thailand; 6 Preclinical Toxicity and Efficacy Assessment of Medicines and Chemicals Research Unit, Chulalongkorn University, Bangkok, Thailand; 7 Metabolomics for Life Sciences Research Unit, Chulalongkorn University, Bangkok, Thailand

**Keywords:** Metabolomics, Neurological disorders

## Abstract

Oxyresveratrol has been documented benefits for neurodegenerative disease. However, the specific molecular mechanisms and pathways involved is currently limited. This study aimed to investigate the potential neuroprotective mechanisms of oxyresveratrol using rotenone-induced human neuroblastoma SH-SY5Y cytotoxicity. Cells were divided into the following groups: control, rotenone, and oxyresveratrol pre-treated before being exposed to rotenone. Cellular assays were performed to investigate neuroprotective effects of oxyresveratrol. The results showed that 20 μM oxyresveratrol was effective in preventing rotenone-induced cell death and decreasing ROS levels in the cells. The alteration of metabolites and pathways involved in the neuroprotective activities of oxyresveratrol were further investigated using LC-QTOF-MS/MS untargeted metabolomics approach. We hypothesized that oxyresveratrol's neuroprotective effects would be associated with neurodegenerative pathways. A total of 294 metabolites were identified. 7,8-dihydrobiopterin exhibited the highest VIP scores (VIP > 3.0; *p* < 0.05), thus considered a biomarker in this study. Our results demonstrated that pretreatment with oxyresveratrol upregulated the level of 7,8-dihydrobiopterin compared to the positive control. Pathway analysis verified that 7,8-dihydrobiopterin was primarily associated with phenylalanine, tyrosine, and tryptophan metabolism (impact = 1, *p* < 0.001), serving as essential cofactors for enzymatic function in the dopamine biosynthesis pathway. In conclusion, oxyresveratrol may be benefit for the prevention of neurodegenerative diseases by increasing 7,8-dihydrobiopterin concentration.

## Introduction

The world’s ageing population is growing rapidly, with the number of people aged 60 and over expected to total more than 2 billion by 2050^1^. In the brain, ageing is associated with a range of structural and functional changes that can increase the risk of neurodegenerative diseases such as Alzheimer’s disease (AD) and Parkinson’s disease (PD)^2^. One of the mechanisms that has been implicated in the pathogenesis of neurodegenerative disease is oxidative stress^3,4^**.** Oxidative stress is the result of an imbalance between the production of reactive oxygen species (ROS) and the ability of the body to counteract their harmful effects. It is thought to be a key contributor to the accumulation of cellular damage that occurs with age. Furthermore, it can induce protein structural and functional change, a decrease in antioxidant enzyme activities, an increase of ROS generation, and DNA damage leading to mitochondrial dysfunction, resulting in neuron cell death^5–7^.

Although neurodegenerative diseases like AD or PD are not currently curable, several studies show that some bioactive substances are linked with a lower risk of developing PD^8,9^. Oxyresveratrol is a structural analog of *trans*-resveratrol, with an additional hydroxyl group attached to one of the aromatic rings^10^, which makes it more water-soluble than *trans*-resveratrol^11^. Oxyresveratrol has demonstrated an antioxidant activity, particularly in its ability to scavenge free radicals^12^. Additionally, oxyresveratrol has been reported to improve mitochondrial dysfunction^13^, decrease lipid peroxidation release, reduce the levels of lactate dehydrogenase, decrease caspase-3 activity, and reduce ROS production induced by 6-OHDA in SH-SY5Y cells^14^. These findings suggest that oxyresveratrol has potential to be a neuroprotective agent. Although oxyresveratrol has demonstrated beneficial effects in various studies, the specific molecular mechanisms underlying these effects remain limited. Further research including identification of the molecules or biological pathways that are targeted by oxyresveratrol is required for comprehensively understanding the specific mechanisms responsible for its neuroprotective effects.

Metabolomics is the study of the complete set of metabolites, the small molecules involved in cellular metabolism^15^. Today, metabolomics is the most advanced technique that sheds light on cellular metabolism. Metabolomic studies on neuron cells can provide valuable information on cellular processes and pathways, including energy metabolism^16^, neurotransmitter biosynthesis^17^, and oxidative stress^18^. It can also help to identify metabolic changes associated with various neurological disorders, such as AD and PD.

The objective of this study was to investigate the potential neuroprotective mechanisms by oxyresveratrol. Rotenone-induced neurotoxicity in human neuroblastoma SH-SY5Y cells was used as a model in this study. We initially examined the optimal concentration of oxyresveratrol from the cellular assays, followed by metabolomics analysis by liquid chromatography quadrupole time-of-flight mass spectrometry (LC-QTOF-MS/MS). This approach allows for the identification and quantification of a large number of metabolites simultaneously, providing a comprehensive view of the cellular metabolic network from complex biological matrices. By identifying the specific metabolites and metabolic pathways, it may be possible to gain insights into the mechanisms underlying oxyresveratrol’s neuroprotective effect. This information could potentially lead to the development of oxyresveratrol as a novel dietary supplement to prevent neurodegenerative diseases in our ageing society.

## Methods

### Chemicals and reagents

Oxyresveratrol, molecular weight 244.24, purity > 98.87%, solubility 50 mg/mL in DMSO, was purchased from MedChem Express (Monmouth Junction, NJ, USA). Rotenone, molecular weight 394.41, purity > 95%, solubility 0.5 mg/mL in DMSO, dimethyl sulfoxide (DMSO), and 7,8-dihydrobiopterin were obtained from Sigma-Aldrich (St. Louis, MO, USA). Phosphate-buffered saline (PBS) was obtained from Merck (Darmstadt, Germany). Dulbecco's Modified Eagle Medium/Nutrient Mixture F-12 (DMEM/F-12), Fetal bovine serum (FBS), and penicillin-streptomycin were obtained from Gibco (Grand Island, NY, USA). SH-SY5Y human neuroblastoma cell line ATCC CRL-2266 was obtained from the American Type Culture Collection (ATCC) (Manassas, VA, USA). 3-(4,5-dimethylthiazol-2-yl)-2,5-diphenyltetrazolium bromide (MTT) and 2′,7′-dichlorodihydrofluorescein diacetate (H_2_DCF-DA) were obtained from Invitrogen (Thermo Fisher Scientific Inc. (Waltham, MA, USA). Primary antibodies including Bax (#2772), Bcl-2 (124) (#15071), cytochrome c (D18C7) (#11940), and β-actin (13E5) (#4967), as well as secondary antibodies anti-rabbit IgG, HRP-linked antibody (#7074) were obtained from Cell Signaling Technology Inc. (Danvers, MA, USA). Acetonitrile and methanol HPLC grade solvents were purchased from Merck (Darmstadt, Germany).

### Cellular assays

#### Cytotoxicity of oxyresveratrol and rotenone on cell viability of SH-SY5Y cells

SH-SY5Y cells (passage ≤ 20) were cultured in DMEM/F12 containing 10% FBS and 1% penicillin and streptomycin, incubated in a 5% CO_2_ humidified incubator at 37 °C. Cells were seeded in the 24-well plates at a density of 5 × 10^5^ cells/well. After incubation for 24 h, cells were treated with 0.5% DMSO and acted as control. To select the half-maximal inhibitory concentration (IC_50_) of rotenone, cells were induced with rotenone (1, 5, 10, 15, and 20 µM) for 48 h. To determine the non-toxic concentration of oxyresveratrol, cells were incubated with oxyresveratrol (5, 10, 20, 50, and 100 µM) for 24 h. Then, the cell viability was determined by MTT assay using a microplate reader (SpectraMax iD5, Molecular Devices, San Jose, CA, USA) at 470 nm. The experiments were carried out in four replicates. Results are calculated as a percentage of control using GraphPad Prism version 8.0.2.

#### Protective effect of oxyresveratrol on rotenone-induced cytotoxicity in SH-SY5Y cells

SH-SY5Y cells were seeded into 24-well plates at a density of 5 × 10^5^ cells per well. Cells were allocated into four groups: control, rotenone (R) (IC_50_), oxyresveratrol (OXY) pre-treated group at 10 µM for 24 h before being exposed to rotenone for 48 h, and oxyresveratrol pre-treated group at 20 µM for 24 h before being exposed to rotenone for 48 h. Then, the cell viability was determined by MTT assay using a microplate reader (SpectraMax iD5, Molecular Devices, San Jose, CA, USA) at 470 nm. The experiments were carried out in four replicates. Results are calculated as a percentage of control using GraphPad Prism version 8.0.2.

#### Measurement of mitochondrial membrane potential (MMP)

The mitochondrial membrane potential was measured by TMRE assay. SH-SY5Y cells were seeded into 96-well plates with a density of 5 × 10^4^ cells/well. After being treated as mentioned above, the plate was washed with PBS and incubated with 100 nM TMRE for 15 min. The fluorescent intensity was measured by a microplate reader at an excitation wavelength of 585 mm and an emission wavelength of 535 nm.

#### Intracellular ROS levels assay

SH-SY5Y cells were seeded into 96-well black plates at a density of 5 × 10^4^ cells/well and allocated to the four treatment groups as mentioned above. After the treatment, the cells were washed with PBS and 10 µM of H_2_DCF-DA was added, dissolved in a serum-free medium. The plate was incubated in the dark for 30 min at 37 °C. Finally, the supernatant was removed and 100 µL of PBS was added. The levels of ROS were determined using a microplate reader (SpectraMax iD5, Molecular devices, San Jose, CA, USA) at excitation 485 and emission 525 nm.

#### Glutathione (GSH) colorimetric detection assay

SH-SY5Y cells were seeded at 2 × 10^6^ cells/well in the 6-well plates. After 24 h for incubation, the cells were allocated into four groups and treated as mentioned above. A GSH colorimetric detection kit (#EIAGSHC, Thermo Fisher Scientific Inc., Waltham, MA, USA) was used to measure GSH content as described in the kit protocol. Briefly, the treated cells were washed with cold PBS and 5% cold salicylic was added. The lysed cells were then centrifuged at 14,000 rpm for 10 min at 4 °C. The supernatant was transferred into a 1.5-mL Eppendorf tube and diluted by 4 volumes of assay buffer, followed by measurement of the absorbance at 405 nm in a microplate reader (SpectraMax iD5, Molecular Devices, San Jose, CA, USA).

#### Western blot analysis

SH-SY5Y cells were seeded at 2 × 10^6^ cells/well in the 6-well plates. After 24 h for incubation, the cells were treated as mentioned above. After lysis, the treated cells in the 6-well plate were incubated for 30 min at 4 °C, and then centrifuged at 16,000 rpm at 4 °C for 5 min. The supernatant was collected for protein determination by Bicinchoninic acid (BCA) assay. For protein analysis, 40 µg of each protein sample was separated by 10% sodium dodecyl sulfate polyacrylamide gel electrophoresis and subsequently transferred to a nitrocellulose membrane. The membrane was blocked with 5% dry milk to eliminate non-specific binding of proteins. The membrane was then incubated with primary antibodies directed against Bax (1:1000), Bcl-2 (1:1000), cytochrome C (1:2000), or β-actin (1:20,000) overnight at 4 °C. Following the incubation period, the membrane was washed with 1X Tris buffered saline-Tween 20 and incubated with a species-specific horseradish peroxidase-conjugated secondary antibody for 2 h. Finally, the antibody-bound proteins were detected using chemiluminescence and quantified using Image J software. β-actin was used as a control for protein expression, and the intensity of selected bands was presented as the relative intensity of proteins and β-actin.

#### Caspase-3 activity assay

SH-SY5Y cells were seeded at 2 × 10^6^ cells/well in a 6-well culture plate. After 24 h for incubation, the cells were treated as mentioned above. To determine caspase-3 activity, the EnzChek caspase-3 assay kit (Molecular Probes, Eugene, OR, USA) was utilized in accordance with the manufacturer's instructions. Briefly, the treated cells were washed with cold PBS and subsequently lysed with lysis buffer for 30 min. The lysed cells were then centrifuged at 5000 rpm for 5 min, and 50 µL of the resulting supernatant was transferred into a 96-well plate. Next, 50 µL of the 2X substrate (Z-DEVD–AMC) working solution was added to each sample and control, and then incubated for 30 min. Sample was then incubated for 30 min at room temperature and measured at excitation 342 and emission 441 nm using a microplate reader (SpectraMax iD5, Molecular Devices, San Jose, CA, USA).

### Statistical analysis

All experiments were carried out in four replicates. Data (mean ± standard deviation) were analyzed by one-way analysis of variance (ANOVA) with Scheffe’s post hoc test using IBM SPSS version 22.0 (IBM SPSS Statistics version, Armonk, NY, USA). A *p*-value < 0.05 was considered significantly different.

### Metabolomics analysis

#### Sample preparation

SH-SY5Y cells were seeded at a density of 2 × 10^6^ cells per well in 6-well plates. Cells were divided into four groups: control, rotenone, pretreatment of oxyresveratrol (OXY20 + R), and oxyresveratrol alone (OXY20). The intracellular metabolites were extracted by washing the cells with cold PBS to remove any remaining culture medium, followed by addition of 80% cold methanol to the cells to quench metabolic activity, followed by a freeze–thaw cycle (alternating between 37 °C and − 80 °C) to lyse the cells and release the intracellular metabolites. The lysate was then transferred to a 1.5-mL tube and centrifuged at 16,000 rpm for 10 min at 4 °C to remove cell debris and collect the supernatant. Supernatants were dried under nitrogen and stored at − 80 °C for subsequent LC-QTOF-MS/MS analysis^17^. The supernatants were dissolved in 200 µL of mobile phase A followed by filtering through an 0.22 µm membrane filter and then transferred to amber glass vials. Eight replicates for each group were analyzed. Meanwhile, 30 μL of each sample were mixed in 1.5-mL tube for the quality control (QC) sample.

#### Data acquisition

Untargeted metabolomics analysis was conducted using a Dionex Ultimate 300 UHPLC system (Thermo Fisher Scientific Inc., Waltham, MA, USA) coupled to QTOF Impact II (Bruker Daltonics, Bremen, Germany). Separation of metabolites was performed on a C18 column (Thermo Fisher Scientific, Sunnyvale, CA, USA) with a particle size of 1.9 µm, 2.1 × 100 mm. Column oven temperature was maintained at 40 °C, while the autosampler was kept at 7 °C. Separation was done by gradient elution of mobile phase A (0.1% formic acid in aqueous solution), and mobile phase B (0.1% formic acid in acetonitrile (v/v)). The following gradient elution conditions were used: 0–99% B over 0 to 15 min, maintained at 99% B for 5 min, followed by 1% B over 20 to 20.1 min, and a final hold at 1% B for 5 min, resulting in a total run time of 25 min. Flow rate was set at 0.3 mL/min.

The acquisition of mass spectra was conducted using electrospray ionization (ESI) in both positive and negative ion modes, covering the mass range of m/z 50–1000. Nitrogen gas was used for the nebulizer and collision gas. The collision energy was 20.0 eV and 10 eV for positive and negative mode, respectively. The dry temperature was 250 °C, and the dry gas flow rate was 8.0 L/min. The capillary voltage was 3800 V and 2500 V for positive and negative mode, respectively.

#### Data processing and statistical analysis

Raw LC–MS/MS data were converted into ABF (analysis base file) format and imported to MS-DIAL version 4.92 for deconvolution, peak detection, peak alignment, and compound identification. Authentic standards, MassBank, GNPS, and Respect (last edited in August 2022) in MSP format were used as the integrated databases for both positive and negative modes. Peak area was then uploaded to MetaboAnalyst 5.0 for statistical analysis. The data were log transformed and pareto scaled. Multivariate analysis was performed by the partial least squares-discriminant analysis (PLS-DA) and candidate biomarkers were collected according to variable importance in projection (VIP) values and the significant differences of each metabolite between groups (*p* < 0.05). Finally, The Human Metabolome Database identifier (HMDB IDs) of the metabolites were uploaded to MetaboAnalyst 5.0 for enrichment and pathway analysis to identify the involved metabolic pathways that were affected by the treatment.

#### Targeted analysis

Metabolites that exhibited high VIP scores were considered as a candidate biomarker. The compound was further analyzed by a targeted approach to confirm its usefulness as an effective biomarker, as suggested by the results of the untargeted analysis in this study. Briefly, another set of SH-SY5Y cells were prepared (n = 4/group). Cells were treated and extracted using the similar method as mentioned above. The targeted analysis of the candidate metabolite was conducted by the same LC-QTOF-MS/MS data acquisition technique, operated in autoMSMS and multiple reaction monitoring (MRM) modes compared to a standard compound (A standard solution was prepared at a concentration of 1 ng/µL by dissolving 7,8-dihydrobiopterin in 50% methanol).

## Results

### Effect of oxyresveratrol and rotenone on cell viability of SH-SY5Y cells

**Figure 1:**
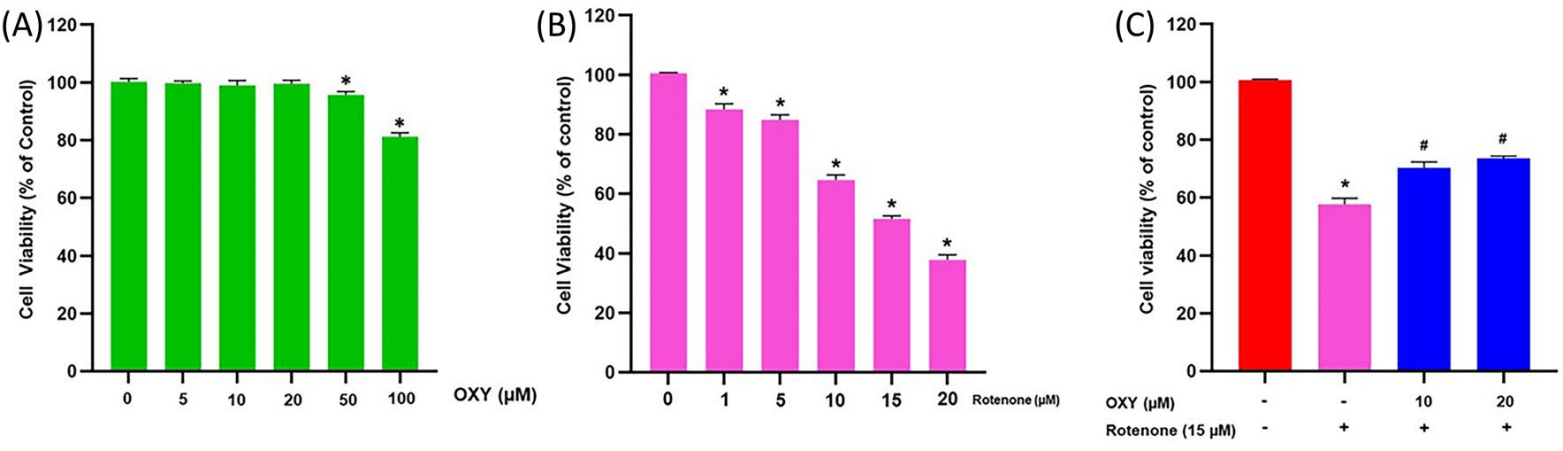
Cytotoxicity assessment in SH-SY5Y cells by MTT assay. (**A**) Various concentrations of oxyresveratrol (0–100 µM). (**B**) Various concentrations of rotenone (0–20 µM). (**C**) Effect of oxyresveratrol on rotenone-induced cytotoxicity in SH-SY5Y cells. Data are expressed as mean ± SD, n = 4. * *p* <  0.05 compared to control, ^**#**^*p* < 0.05 compared to rotenone, degrees of freedom = 3,18 for (**A**) and (**B**) and 3,12 for (**C**). F-value = 388.918, 1352.811, and 98.274 for (**A**), (**B**), and (**C**), respectively.

The MTT assay was conducted to evaluate the cytotoxicity of oxyresveratrol on SH-SY5Y cells. Figure 1A showed that oxyresveratrol 5, 10, and 20 µM did not alter cell viability compared to the control group, implying that oxyresveratrol 20 µM is non-toxic to the cells. Therefore, 20 µM of oxyresveratrol was selected for further experiments. Additionally, the effects of different concentrations of rotenone on cell viability (0, 1, 5, 10, 15, and 20 µM) showed that the IC_50_ of rotenone was 15 µM (Fig. 1B). Therefore, we used 15 µM of rotenone to induce cell death in subsequent experiments.

### Protective effect of oxyresveratrol on rotenone-induced cytotoxicity in SH-SY5Y cells

Rotenone significantly decreased cell viability compared to control (*p* < 0.05; Fig. 1C). The pretreatment groups showed a significant difference compared to the rotenone group with a *p* < 0.05. Treatment with 10 and 20 μM of oxyresveratrol effectively prevented rotenone-induced cell death as exhibited by an increase of approximately 14% and 28%, respectively, compared to the rotenone group.

### Effect of oxyresveratrol on mitochondrial membrane potential

**Figure 2:**
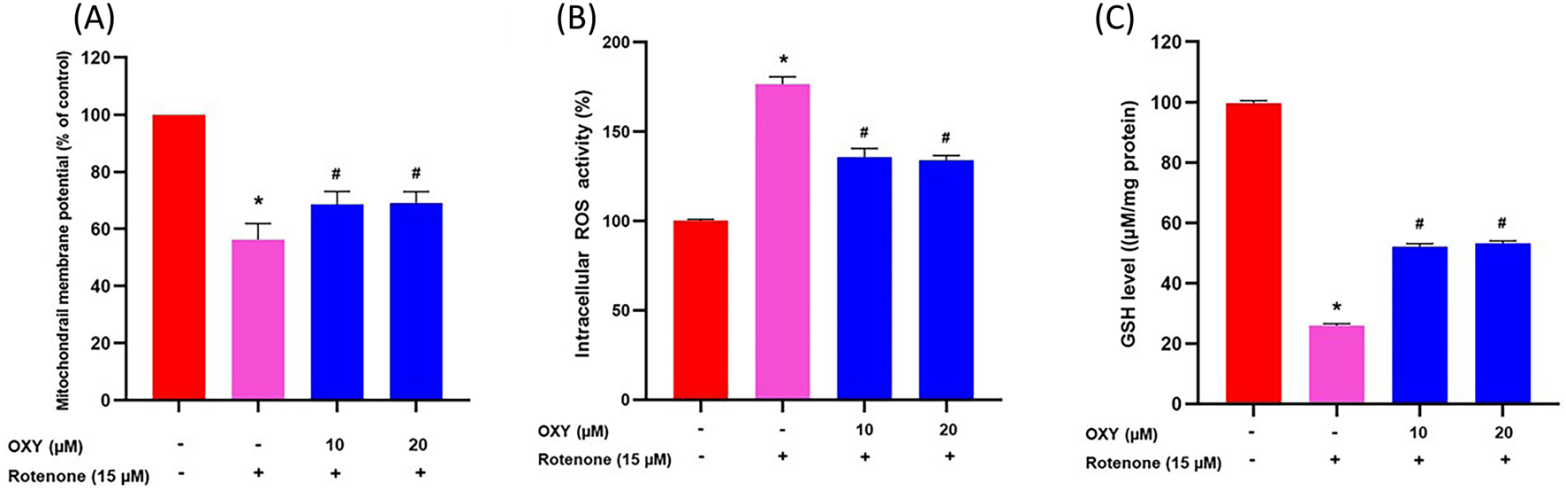
Effect of oxyresveratrol on rotenone-induced MMP, ROS, and GSH levels. (**A**) MMP level, (**B**) ROS generation, (**C**) GSH level. Data are presented as mean ± SD, n = 4. * *p* < 0.05 compared to control, ^**#**^*p* < 0.05 compared to rotenone, degrees of freedom = 3,12. F-value = 61.656, 431.877 and 7548.178 for (**A**), (**B**) and (**C**), respectively.

The results showed that exposure to rotenone resulted in a remarkable reduction in MMP levels (*p* < 0.05). However, when the cells were pre-treated with oxyresveratrol, there was a significant increase in MMP levels as compared to the rotenone group (Fig. 2A).

### Effect of oxyresveratrol on ROS and GSH levels in SH-SY5Y cells treated with rotenone

ROS generation was assessed using H_2_DCF-DA dye in SH-SY5Y cells after exposure to rotenone for 48 h. The results indicated that rotenone exposure increased intracellular ROS levels. However, treatment with 10 and 20 μM of oxyresveratrol resulted in significant decreases in ROS levels compared to the rotenone-exposed group (Fig. 2B). The depletion of GSH levels in SH-SY5Y cells induced by rotenone was reversed by treatment with 10 and 20 μM of oxyresveratrol, as evidenced by significantly increased GSH levels compared to the rotenone-treated group (*p* < 0.05; Fig. 2C). Specifically, GSH levels were increased by 52% and 53%, respectively.

### Effect of oxyresveratrol on apoptosis protein expression in SH-SY5Y cells exposed to rotenone-induced cell death

**Figure 3:**
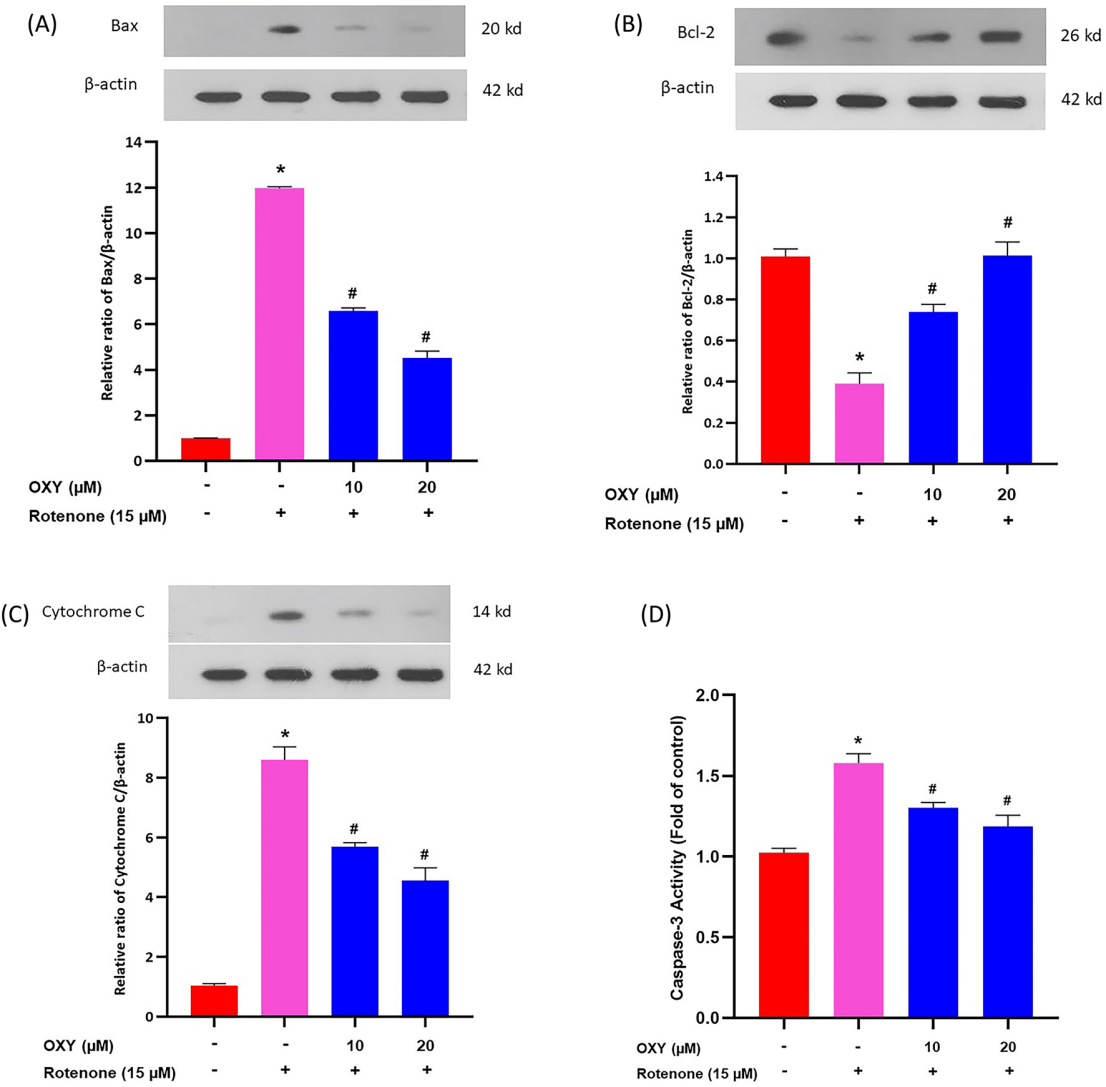
Effect of oxyresveratrol on Bax, Bcl-2, cytochrome C, and caspase-3 expression. Protein levels of (**A**) Bax, (**B**) Bcl-2, and (**C**) Cytochrome C were evaluated by immunoblotting. β-actin immunodetection was used as a loading control. (**D**) Caspase-3 activation. Data are presented as mean ± SD, n = 4. * *p* < 0.05 compared to control, ^**#**^*p* < 0.05 compared to rotenone, degrees of freedom = 3,12. F-value = 11,251.953, 204.992, 37.395, and 59.771 for (**A**), (**B**), (**C**), and (**D**), respectively.

The expression levels of apoptotic proteins were evaluated in SH-SY5Y cells exposed to rotenone, and the results are presented in Fig. 3A–C (Supplementary Fig. S1). Compared to the control group, the rotenone group exhibited higher expression levels of Bax and cytochrome C by 12% and 8% respectively. Oxyresveratrol 10 and 20 μM treatment significantly decreased the expression levels of Bax and cytochrome C compared with rotenone treatment alone (*p* < 0.05). Additionally, Bcl-2 protein expression decreased by 60% in the rotenone group while oxyresveratrol 10 and 20 μM significantly increased Bcl-2 protein expression levels (*p* < 0.05). These findings suggest that treatment of SH-SY5Y cells with oxyresveratrol may enhance the expression of Bcl-2 while decreasing the expression of Bax and cytochrome C proteins.

### Effect of oxyresveratrol on caspase-3 activity in SH-SY5Y cells with induced cell death by rotenone

Caspase-3 activity analysis was used to estimate the level of apoptosis induction in SH-SY5Y cells. According to Fig. 3D, oxyresveratrol was found to reduce apoptosis through a decrease in the caspase-3 activity. The results showed a significant decrease of 25% and 26% in caspase-3 activity in 10 and 20 μM of oxyresveratrol treated and exposed rotenone groups, respectively, compared to the rotenone treated group.

Based on the results of the study, the group treated with 20 μM of oxyresveratrol showed better protection compared to the group treated with 10 μM of oxyresveratrol. It can be pointed out that the higher concentration of oxyresveratrol was more effective in protecting the cells against the damaging effects of rotenone. Therefore, the 20 μM concentration of oxyresveratrol was chosen for further metabolomic analysis as it showed the most promising neuroprotective effects.

### Metabolomics analysis

A total 294 metabolites were identified using the untargeted LC-QTOF-MS/MS based metabolomics approach; 257 and 37 metabolites from the positive and negative mode, respectively (Supplementary Table S1). The metabolites identified in this study were classified as MSI level 2 (putative annotated compounds) according to the Metabolomics Standards Initiative^19^. Examples of secondary mass spectra of the identified endogenous metabolites are provided in Supplementary Fig. S2.

**Figure 4:**
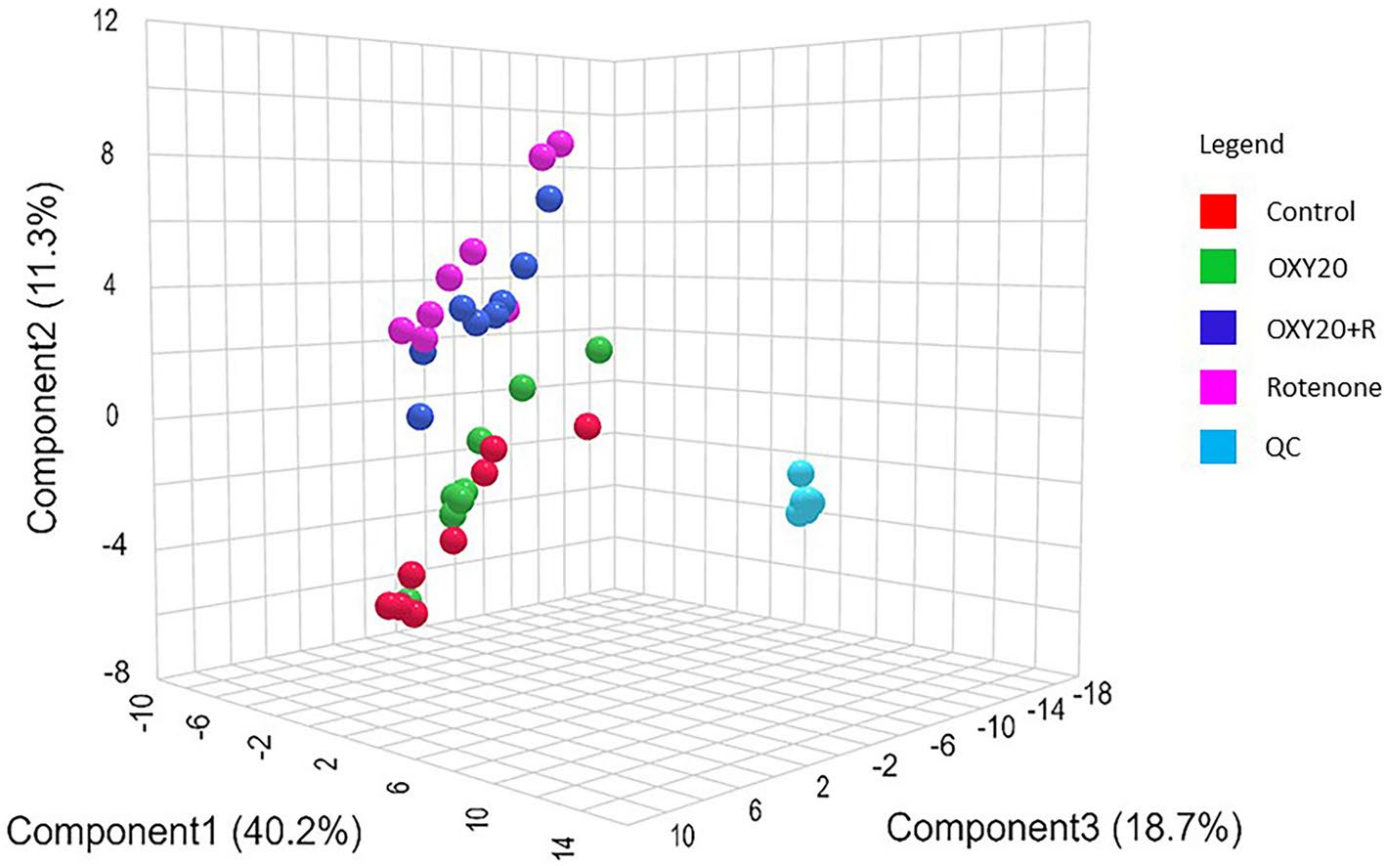
Partial least squares-discriminant analysis (PLS-DA) 3D scores plot of the metabolite dataset. Control (0.5% DMSO), OXY20 (oxyresveratrol pre-treated group at 20 µM for 24 h without exposure to rotenone), OXY20 + R (oxyresveratrol pre-treated group at 20 µM for 24 h and exposed to rotenone (IC_50_) for 48 h), Rotenone (IC_50_), QC (quality control).

From these datasets, the three-dimensional space generated by PLS-DA shows the distribution of analytes from different experimental groups (Fig. 4). Each point represents a sample, colored according to its group membership. The goodness of fit (R^2^) and predictive ability (Q^2^) of the PLS-DA models were 0.98 and 0.75, respectively (Supplementary Fig. S3). The R^2^ value of 0.98 indicates that the PLS-DA model has excellent explanatory power, explaining 98% of the variance in the data. The Q^2^ value of 0.75 indicates that the PLS-DA model has a good ability to predict the behavior of the metabolites. These results imply that the model offers a reliable and robust representation of the underlying structure of the data, enabling dependable predictions to be made. The permutation test for cross-validation (*p* < 0.001) indicates that the observed difference in metabolite profiles between the groups is highly improbable to have occurred by chance, thus indicating its statistical significance (Supplementary Fig. S3).

Considering the metabolomic shifts, the results show that the metabolite profile of the rotenone-treated group is distinctly separated from the control group, indicating that exposure to rotenone caused metabolic changes in the treated cells. The results of the study also revealed that the metabolite profiles of both the OXY and OXY + R groups are closer to the control group than the rotenone group. Notably, pre-treatment with 20 μM of oxyresveratrol resulted in a shift of the OXY + R metabolome towards that of the OXY and control groups.

**Figure 5:**
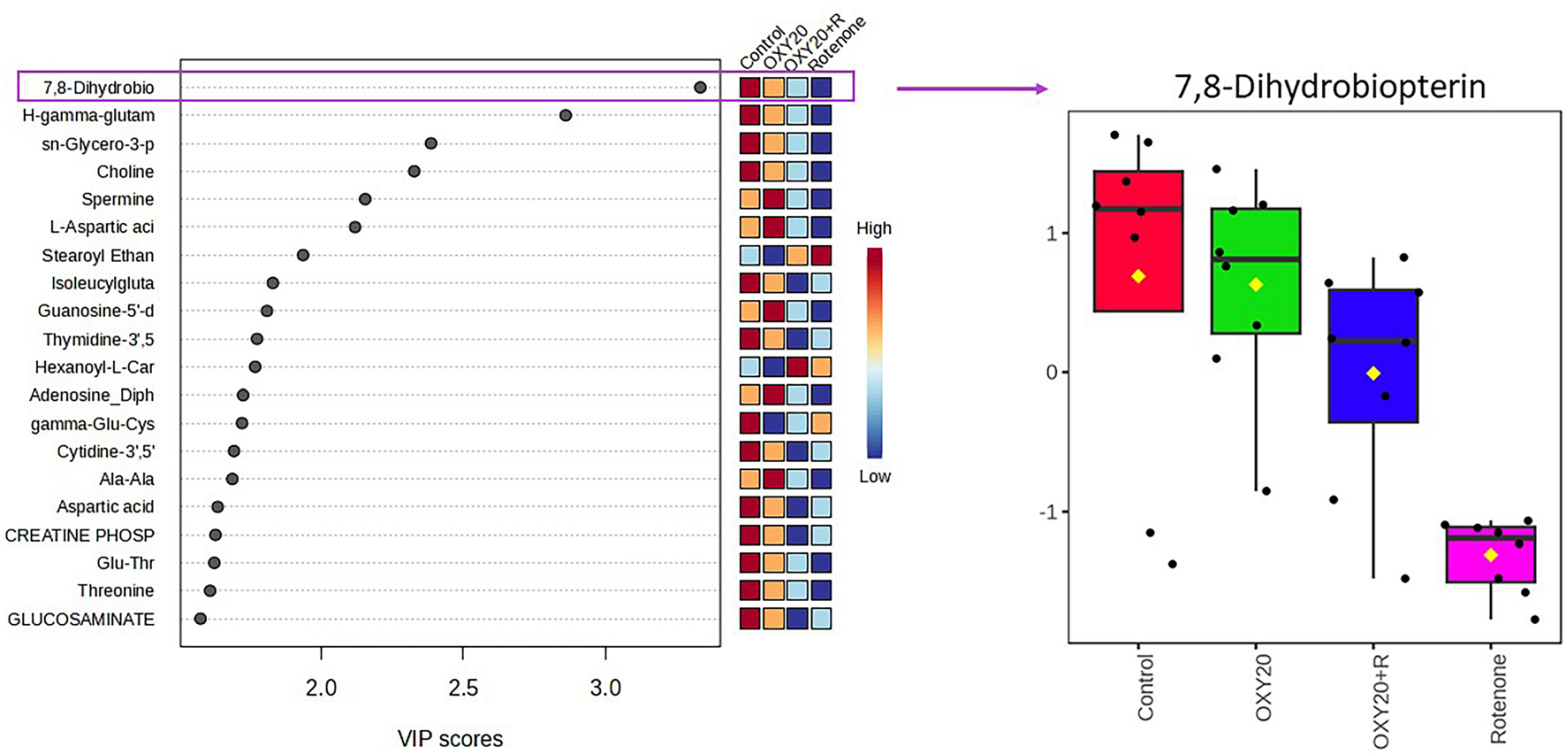
Variable importance in projection (VIP) scores plot of the metabolites dataset with boxplots showing up/down regulation of 7,8-dihydrobiopterin (the metabolite with the highest VIP score). Control (0.5% DMSO), OXY20 (oxyresveratrol pre-treated group at 20 µM for 24 h without exposure to rotenone), OXY20 + R (oxyresveratrol pre-treated group at 20 µM for 24 h and exposed to rotenone (IC_50_) for 48 h), and rotenone (IC_50_).

The identified metabolites were then analyzed using VIP scores, which assess the importance of each metabolite in explaining the differences among the experimental conditions. The VIP value provides a measure of the importance of each metabolite in the separation between different sample groups, with higher VIP values indicating greater importance. Out of the 294 metabolites, 28 compounds had VIP scores greater than 1.5 (Table 1), a typical cutoff point that is frequently used in many studies, suggesting a noticeable impact on the separation between groups. Moreover, among these 28 metabolites, 7,8-dihydrobiopterin exhibited the highest VIP scores (VIP > 3.0; *p* < 0.05) (Fig. 5), indicating that this metabolite had the highest importance in explaining the differences among the experimental conditions. Boxplot of 7,8-dihydrobiopterin also provides for a visual representation of the distribution of this compound across the four distinct experimental conditions. Each boxplot shows the range of values of the metabolite in each experimental condition, with the median value represented by a horizontal line within the box. Findings from the boxplots indicate that the R group had the lowest levels of 7,8-dihydrobiopterin compared to the other experimental conditions, suggesting a potential impact of rotenone exposure on the metabolism related to this compound. Conversely, the OXY group demonstrated higher levels of the metabolites that returned towards those observed in the control group, implying the efficacy of oxyresveratrol pretreatment in mitigating the effects of rotenone exposure on the metabolism.

**Table 1:** List of the metabolites with VIP scores > 1.5 in SH-SY5Y cells pre-treated with oxyresveratrol and exposed to rotenone-induced cell death.

HMDB ID	Metabolites	VIP Scores	Detected m/z	Mass error (ppm)	RT (min)	Adduct	Formula
HMDB0000038	7,8-Dihydrobiopterin	3.3336	240.10667	6.83	1.667	[M + H]^+^	C_9_H_13_N_5_O_3_
HMDB0011738	Ɣ-Glutamyl-glutamine	2.8599	276.11771	3.19	1.125	[M + H]^+^	C_10_H_17_N_3_O_6_
HMDB0000086	Glycerophosphocholine	2.3864	258.10568	19.18	1.002	[M + H] +	C_8_H_21_NO_6_P
HMDB0000097	Choline	2.3272	104.10577	6.53	0.969	[M]^+^	C_5_H_14_NO
HMDB0001256	Spermine	2.1549	203.22141	− 6.94	0.760	[M + H]^+^	C_10_H_26_N_4_
HMDB0000191	L-Aspartic acid	2.1191	134.04361	8.5	1.109	[M + H]^+^	C_4_H_7_NO_4_
HMDB0013078	Stearoylethanolamide	1.9363	328.31665	10.23	15.29	[M + H]^+^	C_20_H_41_NO_2_
HMDB0028906	Isoleucyl-glutamate	1.8299	261.14087	13.1	4.031	[M + H]^+^	C_11_H_20_N_2_O_5_
HMDB0001201	Guanosine diphosphate	1.8093	444.02765	5.29	1.073	[M + H]^+^	C_10_H_15_N_5_O_11_P_2_
HMDB0001570	Thymidine 3',5'-cyclic monophosphate	1.7742	303.04092	− 7.13	1.814	[M − H]^−^	C_10_H_13_N_2_O_7_P
HMDB0000756	Hexanoylcarnitine	1.7678	260.18091	18.06	5.775	[M]^+^	C_13_H_25_NO_4_
HMDB0247869	Adenosine_diphosphate	1.7253	428.03293	10.33	1.081	[M + H]^+^	C_10_H_15_N_5_O_10_P_2_
HMDB0001049	Ɣ-Glutamylcysteine	1.7214	251.06676	11.43	2.386	[M + H]^+^	C_8_H_14_N_2_O_5_S
HMDB0011691	Cytidine 2',3'-cyclic phosphate	1.6933	304.03653	− 8.32	1.372	[M − H]^−^	C_9_H_12_N_3_O_7_P
HMDB0003459	D-Alanyl-D-alanine	1.6874	161.09023	7.26	1.536	[M + H]^+^	C_6_H_12_N_2_O_3_
HMDB0006483	D-Aspartic acid	1.6359	134.04225	18.87	1.367	[M + H]^+^	C_4_H_7_NO_4_
HMDB0001511	Phosphocreatine	1.6281	212.04012	14.01	1.020	[M + H]^+^	C_4_H_10_N_3_O_5_P
HMDB0028829	Glutamylthreonine	1.6240	249.10522	11.16	1.130	[M + H]^+^	C_9_H_16_N_2_O_6_
HMDB0000167	L-Threonine	1.6093	120.06397	8.58	1.130	[M + H]^+^	C_4_H_9_NO_3_
HMDB0341308	D-Glucosaminic acid	1.5756	196.08104	1.38	1.688	[M + H]^+^	C_6_H_13_NO_6_
HMDB0004483	Estrone glucuronide	1.5718	445.18240	9.88	9.077	[M − H]^-^	C_24_H_30_O_8_
HMDB0000791	Octanoylcarnitine	1.5697	288.21350	11.76	7.568	[M + H]^+^	C_15_H_29_NO_4_
HMDB0029737	Indole-3-carboxaldehyde	1.5668	144.04648	− 6.87	1.638	[M − H]^−^	C_9_H_7_NO
HMDB0000064	Creatine	1.5493	132.07494	13.7	1.015	[M + H]^+^	C_4_H_9_N_3_O_2_
HMDB0001553	2-Oxo-4-methylthiobutanoic acid	1.5483	147.00986	7.01	1.395	[M − H]^−^	C_5_H_8_O_3_S
HMDB0013222	Beta-Guanidinopropionic acid	1.5143	132.07494	10.22	1.015	[M + H]^+^	C_4_H_9_N_3_O_2_
N/A	3-Acetylthiazolidine-4-carboxylic acid	1.5026	176.03572	24.26	2.058	[M + H]^+^	C_6_H_9_NO_3_S
HMDB0000812	N-Acetyl-L-aspartic acid	1.5024	176.05351	10.39	1.369	[M + H]^+^	C_6_H_9_NO_5_

**Figure 6:**
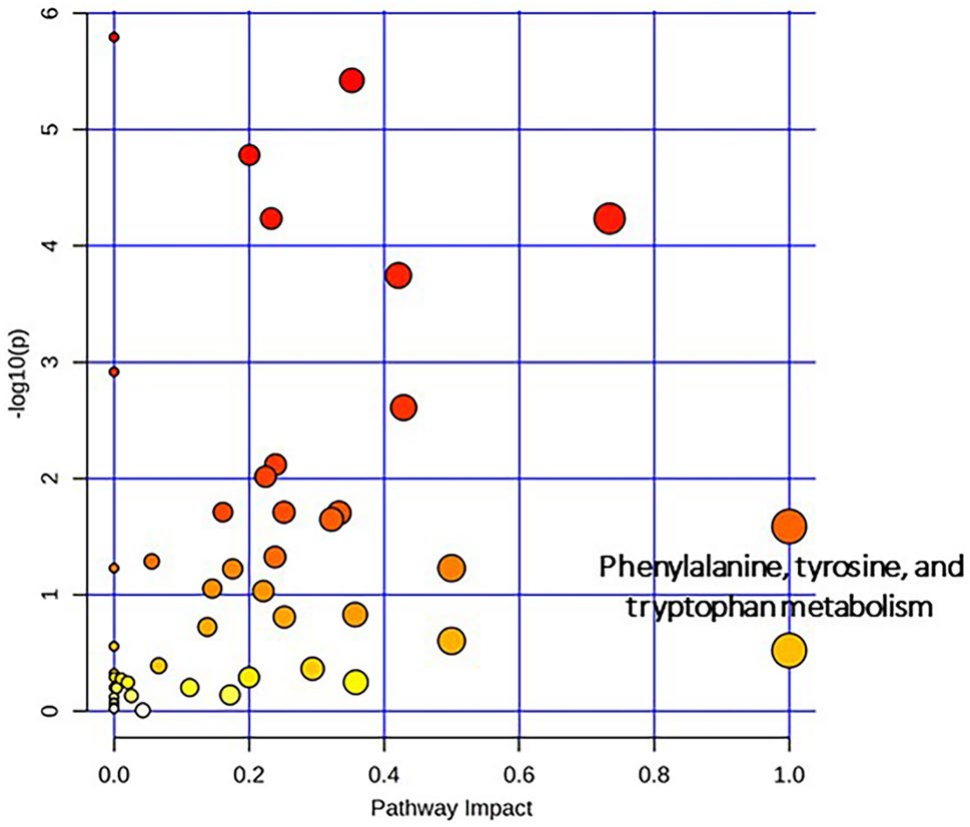
Pathway analysis of SH-SY5Y cells pre-treated with oxyresveratrol and exposed to rotenone-induced cell death.

Metabolic pathways related to neuroprotective activities of oxyresveratrol against rotenone-induced toxicity were investigated in this study by MetaboAnalyst 5.0 software. Several metabolic pathways were significantly associated with pretreatment of oxyresveratrol in rotenone-induced human neuroblastoma SH-SY5Y cytotoxicity. It was found that phenylalanine, tyrosine and tryptophan metabolism exhibited the most impact value (impact value = 1) with *p* < 0.05 (Fig. 6, Supplementary Table S2).

**Figure 7:**
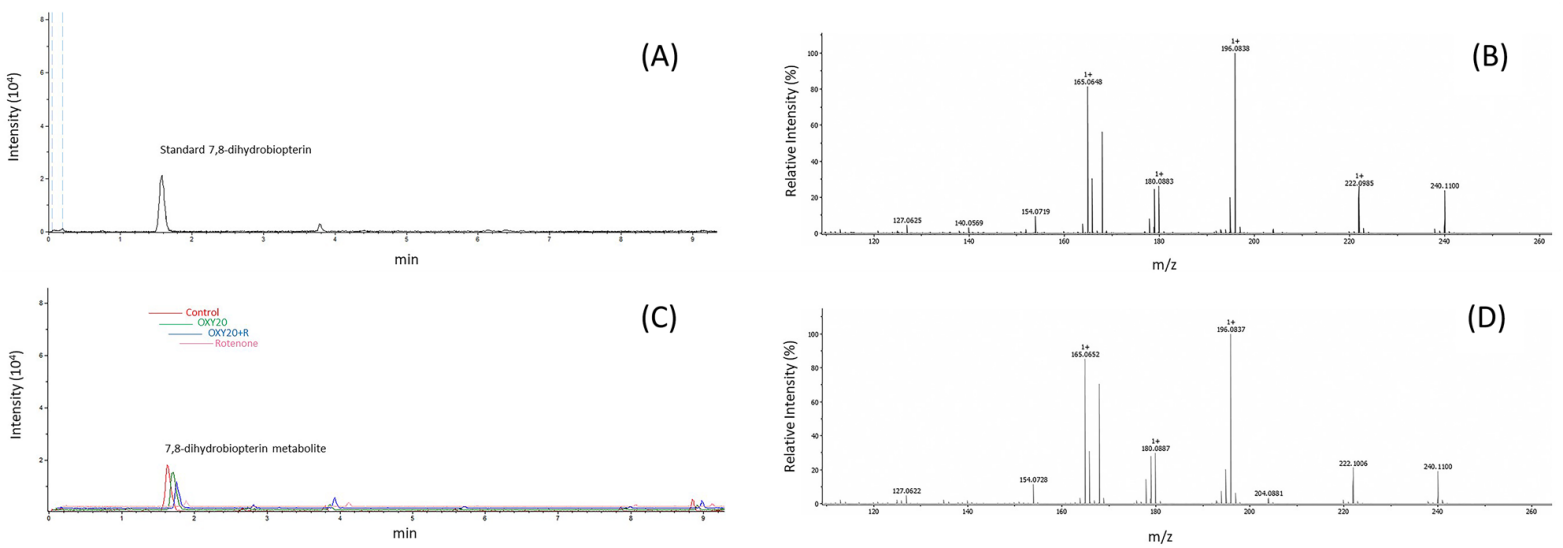
Extracted ion chromatogram (EIC) and Multiple reaction monitoring (MRM) LC-QTOF-MS/MS of 7,8-dihydrobiopterin. (**A**) EIC of standard 7,8-dihydrobiopterin (1 ng/µL). (**B**) Secondary mass spectra of standard 7,8-dihydrobiopterin (1 ng/µL). (**C**) EIC of the 7,8-dihydrobiopterin metabolite in control sample. (**D**) Secondary mass spectra of 7,8-dihydrobiopterin metabolite in control sample.

To support the outcome of the untargeted metabolomics study, 7,8-dihydrobiopterin concentration was subsequently examined using a targeted metabolomics approach with autoMSMS and MRM modes. The extracted ion chromatogram (EIC) from the autoMSMS mode showed that the intensity of the OXY20 + R group was significantly increased compared to the rotenone group (*p* < 0.001, Supplementary Table S3). MRM is a highly selective mode dedicated to monitoring specific transitions between precursor and product ions. In this study, it is employed to selectively detect and quantify 7,8-dihydrobiopterin and its related ions to confirm the presence of the metabolite identified in the untargeted study (Fig. 7, Supplementary Fig. S4).

## Discussion

In this study, we investigated the potential neuroprotective effects of oxyresveratrol in human neuroblastoma SH-SY5Y cells exposed to rotenone, a known initiator of cell death. The SH-SY5Y neuroblastoma cell line is a commonly utilized as an in vitro model for PD research. These cells express several key proteins that are important for dopaminergic neuron function, including dopamine transporters and receptors^20^. Rotenone is often used as a tool to investigate the effects of mitochondrial dysfunction due to its ability to inhibit complex I of the mitochondrial respiratory chain. This inhibition results in decreased ATP production and increased oxidative stress, leading to the release of pro-apoptotic factors, including cytochrome C, and the initiation of apoptosis^21–23^. Rotenone is a lipophilic compound that can easily cross cell membranes and accumulate in the mitochondria. Exposure to rotenone has been associated with the pathogenesis of several diseases, including PD, due to its neurotoxic effects^20,24,25^. A previous study has reported that rotenone induced cytotoxicity in HepG2 cells through several mechanisms, including the generation of ROS, depletion of intracellular GSH, and induction of mitochondria-mediated apoptosis involving p53, Bax/Bcl-2, and caspase-3^26^.

Oxyresveratrol can protect against rotenone-induced toxicity through its ability to promote cell survival. In the present study, the results show that rotenone exposure induces cell death by decreasing MMP levels and generating ROS, a highly reactive molecule causes oxidative damage to cellular components. In consideration, treatment with oxyresveratrol appears to mitigate this effect by increasing MMP levels, reducing ROS generation and increasing the production of GSH, a key antioxidant that protects cells from oxidative damage by scavenging free radical ROS that can damage cell membranes, proteins, and DNA^27^.

Bax, Bcl-2, and cytochrome C are key proteins that regulate the apoptosis (programmed cell death) process in the mitochondria. The expression of Bax results in the release of cytochrome C from mitochondria, which leads to the apoptosis process. On the other hand, the expression of Bcl-2 prevents the release of cytochrome C, thereby preventing the induction of apoptosis^28^. In the present study, the results demonstrated that rotenone significantly stimulated the expression of Bax and decreased the expression of Bcl-2 proteins in SH-SY5Y. In addition, rotenone activated the release of cytochrome C from the mitochondria into the cytosol, which could activate the caspase-3 enzyme^26^. Caspase-3 is a cysteinyl aspartate-specific protease and an initiator caspase that plays an important role in the execution phase of apoptosis^29^. The present study has indicated that oxyresveratrol can increase the expression of the anti-apoptotic protein Bcl-2 and decrease the expression of the pro-apoptotic protein Bax. This shift in the balance between Bcl-2 and Bax can potentially inhibit apoptosis and promote cell survival. It has also been demonstrated that oxyresveratrol can decrease the expression of cytochrome C. By decreasing cytochrome C expression, oxyresveratrol can potentially inhibit caspase-3 activity.

According to the results obtained from the metabolomic analysis, as indicated by the PLS-DA scores plot, pretreatment with oxyresveratrol at a concentration of 20 μM could potentially provide a beneficial effect on the cellular metabolome. The findings suggested that oxyresveratrol may be effective in preventing metabolic changes induced by rotenone (Fig. 4). In this study, 7,8-dihydrobiopterin was identified as a potential biomarker with VIP > 3.0, a score obtained from the PLS-DA model that suggests the potential of this metabolite as a biomarker (Fig. 5). Our results demonstrated that pretreatment with oxyresveratrol upregulated the level of 7,8-dihydrobiopterin compared to the group only exposed to rotenone. Pathway analysis also revealed that phenylalanine, tyrosine, and tryptophan biosynthesis was the most relevant metabolism associated with neuroprotective effects of oxyresveratrol against rotenone-induced toxicity (Fig. 6). The compound 7,8-dihydrobiopterin (BH2) is an intermediary molecule in the biosynthesis of the cofactor tetrahydrobiopterin (BH4)^30^. This cofactor is essential for the function of enzymes that catalyze the hydroxylation of amino acids, specifically phenylalanine and tryptophan, which are important for the synthesis of dopamine and serotonin in nervous systems^17^.

**Figure 8:**
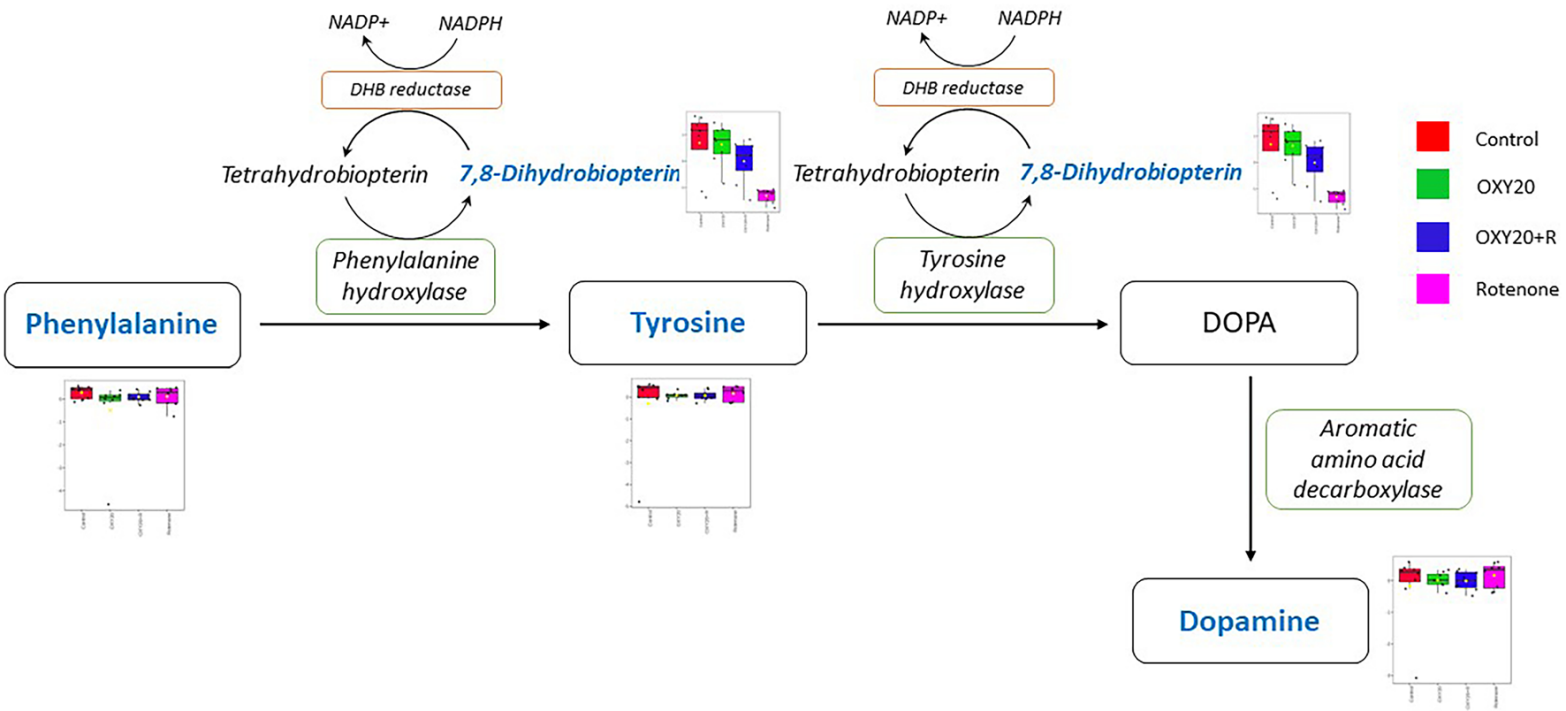
Effect of oxyresveratrol on dopamine biosynthesis pathway in SH-SY5Y cells induced by rotenone. DOPA: 3,4-Dihydroxyphenylalanine, NADPH: Nicotinamide adenine dinucleotide phosphate, DHB: Dihydropteridine. Metabolites (in blue color) with boxplots are also identified by LC-QTOF-MS/MS in this study.

Thus, from untargeted metabolomics analysis in the present study, we hypothesized that oxyresveratrol could protect dopaminergic neurons and prevent their death through the dopamine biosynthesis pathway, as illustrated in Fig. 8. The primary metabolic pathway for phenylalanine involves its conversion to tyrosine through an enzyme called phenylalanine hydroxylase. This process is essential because tyrosine serves as a precursor for several important molecules, including neurotransmitters like dopamine and norepinephrine^31^. Tyrosine is then converted to 3,4-dihydroxyphenylalanine (DOPA) by the enzyme tyrosine hydroxylase, with the cofactor tetrahydrobiopterin (BH4)^32–34^. Furthermore, 7,8-dihydrobiopterin (BH2) can be converted to BH4 by the dihydropteridine (DHB) reductase enzyme. This conversion is an essential step in maintaining the proper function of both enzymes, phenylalanine hydroxylase, and tyrosine hydroxylase^35^. Subsequently, DOPA is further converted to dopamine by the enzyme aromatic amino acid decarboxylase^36,37^. Dopamine is an important neurotransmitter in the brain, and its dysregulation is associated with several neurological conditions, including neurodegenerative disease such as PD^38–40^.

Verification of the key metabolite in this study was carried out by a targeted metabolomics method. Levels of 7,8-dihydrobiopterin (BH2) were further investigated in another set of samples to confirm the novel hypothesis of using this metabolite as a marker for neuroprotective effects by oxyresveratrol. The results verified that 7,8-dihydrobiopterin levels in the OXY + R group were significantly greater than those in the rotenone group, which is in agreement with the results of the untargeted study. Thus, oxyresveratrol pretreatment could elevate levels of the 7,8-dihydrobiopterin metabolite, which may be advantageous when using this compound for preventing neurogenerative diseases.

## Conclusion

The cellular assays show that oxyresveratrol was effective in preventing rotenone-induced cell death and decreasing ROS levels in the cells. An untargeted metabolomics approach provided information regarding the candidate biomarkers and the enriched metabolic pathways, giving valuable new insight into the potential mechanisms underlying the prevention of neurodegenerative diseases by oxyresveratrol. Untargeted metabolomics revealed that 7,8-dihydrobiopterin (BH2) was the key metabolite associated with neuroprotective effects of oxyresveratrol in SH-SY5Y cells. Findings from pathway analysis also supported that 7,8-dihydrobiopterin (BH2), the biomarker from this study, was related to phenylalanine, tyrosine, and tryptophan metabolism, which served as an essential cofactor for the function of enzymes in the dopamine biosynthesis pathway. Therefore, 7,8-dihydrobiopterin (BH2) may be used as an indicator of treatment efficacy, providing a means to monitor the response to oxyresveratrol and evaluate its neuroprotective potential against neurodegenerative diseases. This information could help to guide future research in this area for the development of novel therapeutic approaches for neurodegenerative disease in the elderly.

### Supplementary Information

Supplementary Figure S1.

Supplementary Figure S2.

Supplementary Figure S3.

Supplementary Figure S4.

Supplementary Table S1.

Supplementary Table S2.

Supplementary Table S3.

## Data Availability

All data generated or analyzed during this study are included in this published article as a supplementary information file (Supplementary Table S1).

## Abbreviations

AD: Alzheimer’s disease
DMSO: Dimethyl sulfoxide
DNA: Deoxyribonucleic acid
DOPA: 3,4-Dihydroxyphenylalanine
GSH: Glutathione
IC_50_: Half-maximal inhibitory concentration
LC-QTOF-MS/MS: Liquid chromatography quadrupole time-of-flight mass spectrometry
m/z: Mass to charge ratio
MMP: Mitochondrial membrane potential
OXY: Oxyresveratrol
PD: Parkinson’s disease
PLS-DA: Partial least squares-discriminant analysis
QC: Quality control
R: Rotenone
ROS: Reactive oxygen species
RT: Retention time
VIP: Variable importance in projection

## References

[1] Oliveira JS (2023). Effect of sport on health in people aged 60 years and older: A systematic review with meta-analysis. Br. J. Sports Med..

[2] Moore K, Hughes CF, Ward M, Hoey L, McNulty H (2018). Diet, nutrition and the ageing brain: Current evidence and new directions. Proc. Nutr. Soc..

[3] Tadtong S (2017). Effects of oxyresveratrol and its derivatives on cultured P19-derived neurons. Trop. J. Pharm. Res..

[4] Aborode AT (2022). Targeting oxidative stress mechanisms to treat Alzheimer's and Parkinson's disease: a critical review. Oxid. Med. Cell Longev..

[5] Prakash R (2022). Oxidative stress-induced autophagy compromises stem cell viability. Stem Cells.

[6] Gonzalez-Sarrias A, Nunez-Sanchez MA, Tomas-Barberan FA, Espin JC (2017). Neuroprotective effects of bioavailable polyphenol-derived metabolites against oxidative stress-induced cytotoxicity in human neuroblastoma SH-SY5Y cells. J. Agric. Food Chem..

[7] Singh A, Kukreti R, Saso L, Kukreti S (2019). Oxidative stress: a key modulator in neurodegenerative diseases. Mol.

[8] Luo M (2021). Effects and mechanisms of tea on Parkinson’s disease, Alzheimer’s disease and depression. Food Rev. Int..

[9] Piechowska P, Zawirska-Wojtasiak R, Mildner-Szkudlarz S (2019). Bioactive beta-carbolines in food: A review. Nutrients.

[10] Likhitwitayawuid K (2021). Oxyresveratrol: Sources, productions, biological activities, pharmacokinetics, and delivery systems. Mol.

[11] Chen W, Yeo SCM, Elhennawy MGAA, Lin H-S (2016). Oxyresveratrol: A bioavailable dietary polyphenol. J. Funct. Foods.

[12] Weber JT (2012). Potential neuroprotective effects of oxyresveratrol against traumatic injury. Eur. J. Pharmacol..

[13] Hasriadi WM, Lapphanichayakool P, Limpeanchob N (2017). Neuroprotective effect of Artocarpus Lakoocha extract and oxyresveratrol against hydrogen peroxide-Induced toxicity in SH-SY5Y cells. Int. J. Pharm. Pharm. Sci..

[14] Chao J, Yu MS, Ho YS, Wang M, Chang RC (2008). Dietary oxyresveratrol prevents parkinsonian mimetic 6-hydroxydopamine neurotoxicity. Free Radic. Biol. Med..

[15] Bedia C (2022). Metabolomics in environmental toxicology: Applications and challenges. Trends Environ. Anal. Chem..

[16] Maker GL, Green T, Mullaney I, Trengove RD (2018). Untargeted metabolomic analysis of rat neuroblastoma cells as a model system to study the biochemical effects of the acute administration of methamphetamine. Metabolites.

[17] Zong L, Xing J, Liu S, Liu Z, Song F (2018). Cell metabolomics reveals the neurotoxicity mechanism of cadmium in PC12 cells. Ecotoxicol. Environ. Saf..

[18] Xie Q (2018). Protective effects of timosaponin BII on oxidative stress damage in PC12 cells based on metabolomics. Biomed. Chromatogr..

[19] Kodra D (2022). Is current practice adhering to guidelines proposed for metabolite identification in LC-MS untargeted metabolomics? A meta-analysis of the literature. J. Proteome Res..

[20] Ioghen OC, Ceafalan LC, Popescu BO (2023). SH-SY5Y cell line in vitro models for Parkinson disease research-old practice for new trends. J. Integr. Neurosci..

[21] Kaneko K, Hineno A, Yoshida K, Ikeda S (2008). Increased vulnerability to rotenone-induced neurotoxicity in ceruloplasmin-deficient mice. Neurosci. Lett..

[22] PreetKaur K, Khurana N, Sharma N (2021). Phytochemicals as future drugs for Parkinson’s disease: A review. Plant Arch..

[23] Gonzalez-Burgos E, Fernandez-Moriano C, Lozano R, Iglesias I, Gomez-Serranillos MP (2017). Ginsenosides Rd and Re co-treatments improve rotenone-induced oxidative stress and mitochondrial impairment in SH-SY5Y neuroblastoma cells. Food Chem. Toxicol..

[24] Sanders LH, Timothy Greenamyre J (2013). Oxidative damage to macromolecules in human Parkinson disease and the rotenone model. Free Radic. Biol. Med..

[25] Yarmohammadi F, Wallace Hayes A, Najafi N, Karimi G (2020). The protective effect of natural compounds against rotenone-induced neurotoxicity. J. Biochem. Mol. Toxicol..

[26] Siddiqui MA (2013). Rotenone-induced oxidative stress and apoptosis in human liver HepG2 cells. Mol. Cell Biochem..

[27] Aoyama K (2021). Glutathione in the brain. Int. J. Mol. Sci..

[28] Alam M (2022). Bax/Bcl-2 cascade is regulated by the EGFR pathway: Therapeutic targeting of non-small cell lung cancer. Front. Oncol..

[29] Asadi M (2022). Caspase-3: Structure, function, and biotechnological aspects. Biotechnol. Appl. Biochem..

[30] Hasegawa H, Sawabe K, Nakanishi N, Wakasugi OK (2005). Delivery of exogenous tetrahydrobiopterin (BH4) to cells of target organs: role of salvage pathway and uptake of its precursor in effective elevation of tissue BH4. Mol. Genet. Metab..

[31] Frings C, Domes G, Friehs MA, Geißler C, Schneider K (2019). Food for your mind? The effect of tyrosine on selective attention. J. Cogn. Enhanc..

[32] Nagatsu T, Nakashima A, Ichinose H, Kobayashi K (2019). Human tyrosine hydroxylase in Parkinson's disease and in related disorders. J. Neural Transm..

[33] Flydal MI (2021). Levalbuterol lowers the feedback inhibition by dopamine and delays misfolding and aggregation in tyrosine hydroxylase. Biochimie.

[34] Du D (2022). Biomimetic synthesis of L-DOPA inspired by tyrosine hydroxylase. J. Inorg. Biochem..

[35] Lo A (2017). Single-step rapid diagnosis of dopamine and serotonin metabolism disorders. ACS Omega.

[36] Beckers M, Bloem BR, Verbeek MM (2022). Mechanisms of peripheral levodopa resistance in Parkinson's disease. NPJ Parkinsons Dis..

[37] Rumund AV (2021). Peripheral decarboxylase inhibitors paradoxically induce aromatic L-amino acid decarboxylase. NPJ Parkinsons Dis..

[38] Nutt JG (2020). Aromatic L-amino acid decarboxylase gene therapy enhances levodopa response in Parkinson's disease. Mov. Disord..

[39] Meder D, Herz DM, Rowe JB, Lehericy S, Siebner HR (2019). The role of dopamine in the brain—Lessons learned from Parkinson's disease. Neuroimage.

[40] Klein MO (2019). Dopamine: Functions, signaling, and association with neurological diseases. Cell Mol. Neurobiol..

