# Supplementary material for: Untargeted metabolomics reveal pathways associated with neuroprotective effect of oxyresveratrol in SH-SY5Y cells
Source: Sci Rep. 2023 Nov 21;13:20385. doi: 10.1038/s41598-023-47558-y (PMC10663518; doi:10.1038/s41598-023-47558-y)
Supplement: Supplementary file 1 — Supplementary Figure S1. [file 41598_2023_47558_MOESM1_ESM.pdf]

Untargeted metabolomics reveal pathways associated with neuroprotective effect of oxyresveratrol in SH-SY5Y cells

Nureesun Mahamud[1,2], Phanit Songvut[3], Chawanphat Muangnoi[4], Ratchanee Rodsiri[5,6], Winai Dahlan[2] & Rossarin Tansawat[1,7]*

[1] Department of Food and Pharmaceutical Chemistry, Faculty of Pharmaceutical Sciences, Chulalongkorn University, Bangkok, 10330, Thailand.
[2] The Halal Science Center, Chulalongkorn University, Bangkok, 10330, Thailand.
[3] Laboratory of Pharmacology, Chulabhorn Research Institute, Bangkok, 10210, Thailand.
[4] Cell and Animal Model Unit, Institute of Nutrition, Mahidol University, Nakhon Pathom, 73170, Thailand.
[5] Department of Pharmacology and Physiology, Faculty of Pharmaceutical Sciences, Chulalongkorn University, Bangkok, 10330, Thailand.
[6] Preclinical Toxicity and Efficacy, Assessment of Medicines and Chemicals Research Unit, Chulalongkorn University, Bangkok, 10330, Thailand
[7] Metabolomics for Life Sciences Research Unit, Chulalongkorn University, Bangkok, 10330, Thailand

* Corresponding author: rossarin.t@Pharm.Chula.ac.th

Corresponding author:
Rossarin Tansawat, PhD
Department of Food and Pharmaceutical Chemistry
Faculty of Pharmaceutical Sciences
Chulalongkorn University
254 Phayathai Road, Wangmai, Pathumwan
Bangkok 10330 Thailand
rossarin.t@pharm.chula.ac.th

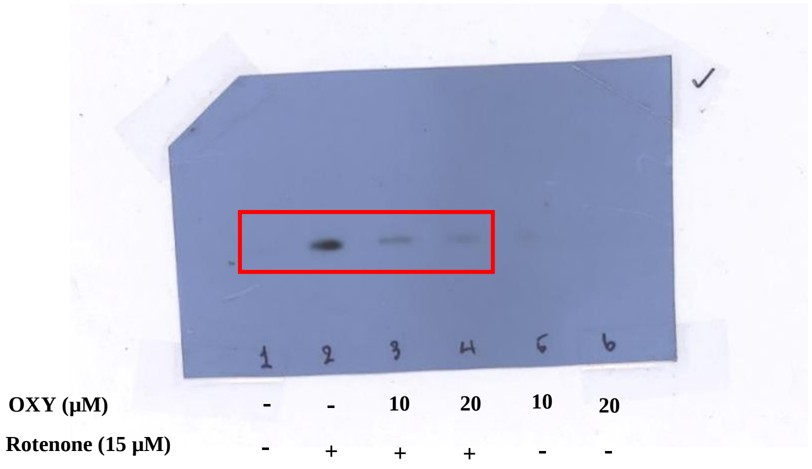

Supplementary Figure S1.1. The original full blots of Bax (Replication 1). Red box indicates the region of the original blot used in main figure.

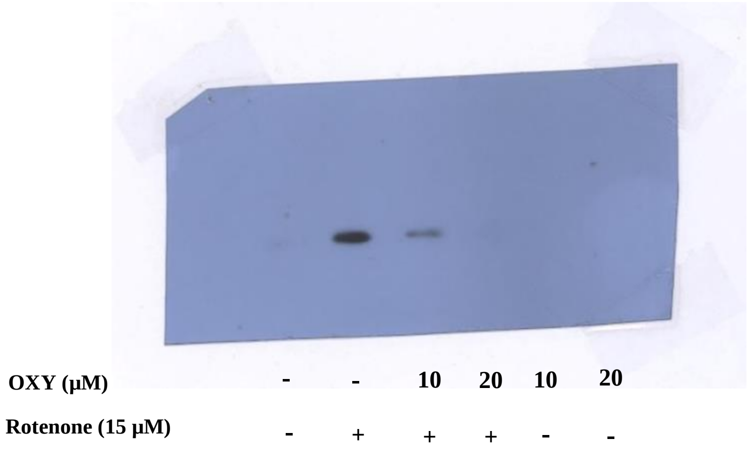

Supplementary Figure S1.2. The original full blots of Bax (Replication 2).

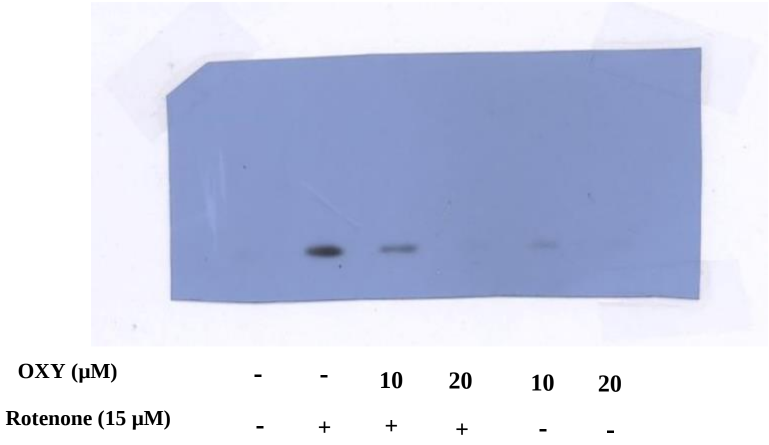

Supplementary Figure S1.3. The original full blots of Bax (Replication 3).

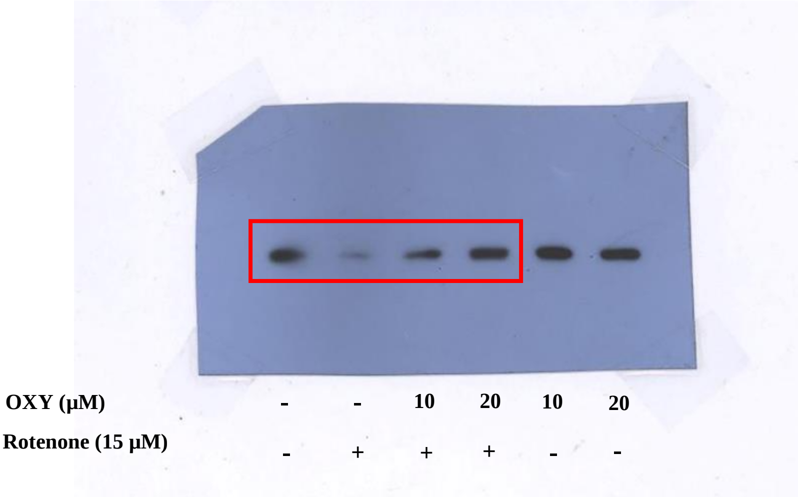

OXY (µM)	-	-	10	20	10	20
Rotenone (15 µM)	-	+	+	+	-	-

Supplementary Figure S1.4. The original full blots of Bcl-2 (Replication 1). Red box indicates the region of the original blot used in main figure.

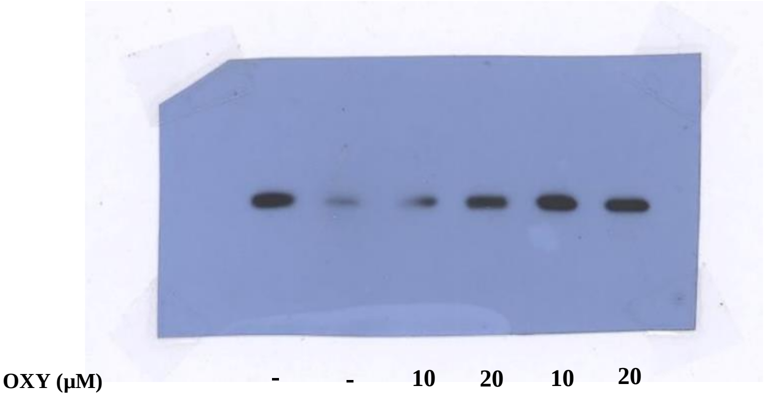

OXY (µM)	-	-	10	20	10	20
Rotenone (15 µM)	-	+	+	+	-	-

Supplementary Figure S1.5. The original full blots of Bcl-2 (Replication 2).

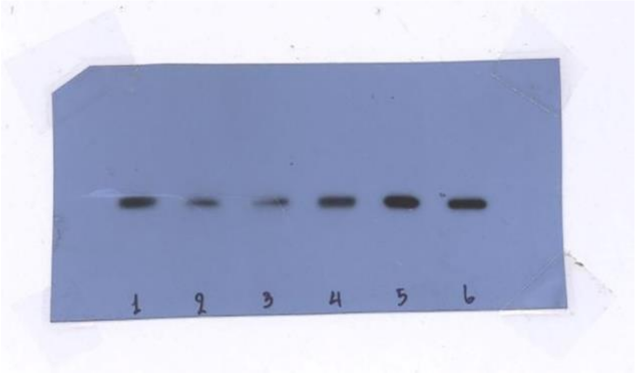

OXY (µM)	-	-	10	20	10	20
Rotenone (15 µM)	-	+	+	+	-	-

Supplementary Figure S1.6. The original full blots of Bcl-2 (Replication 3).

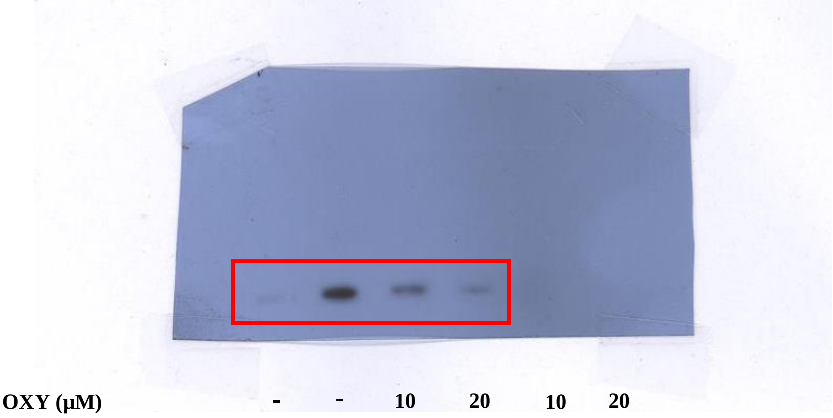

Supplementary Figure S1.7. The original full blots of Cytochrome C (Replication 1). Red box indicates the region of the original blot used in main figure.

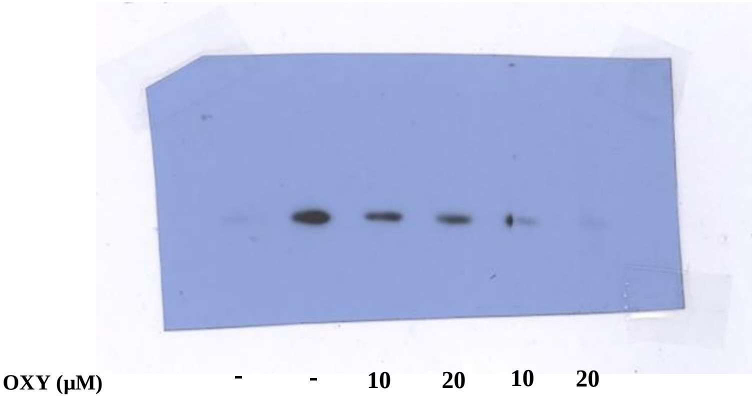

Supplementary Figure S1.8. The original full blots of Cytochrome C (Replication 2).

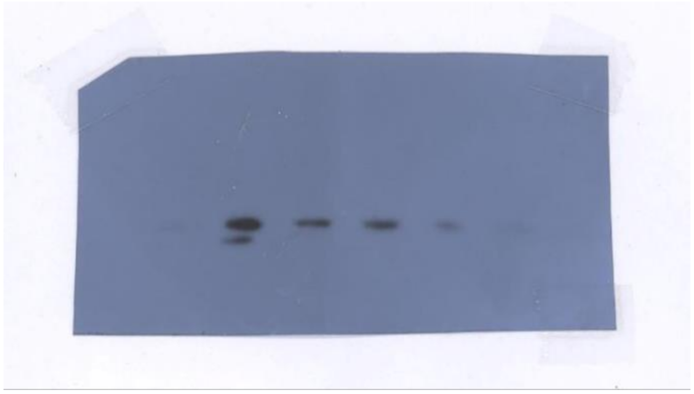

Supplementary Figure S1.9. The original full blots of Cytochrome C (Replication 3).

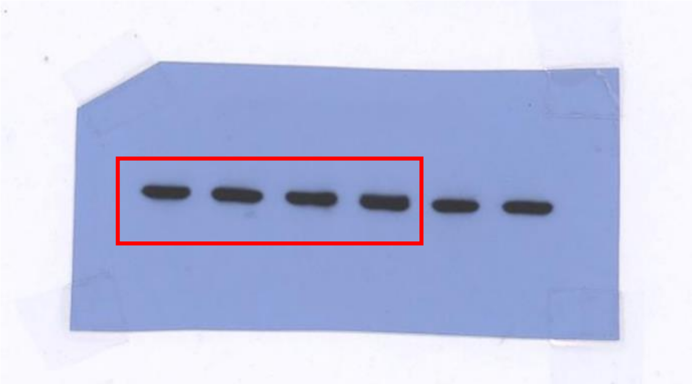

**OXY (μM)**	-	-	10	20	10	20
**Rotenone (15 μM)**	-	+	+	+	-	-

Supplementary Figure S1.10. The original full blots of β-actin for Bax. Red box indicates the region of the original blot used in main figure.

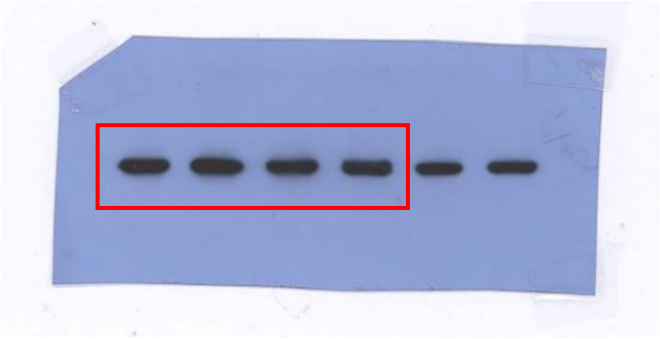

**OXY (μM)**	-	-	10	20	10	20
**Rotenone (15 μM)**	-	+	+	+	-	-

Supplementary Figure S1.11. The original full blots of β-actin for Bcl-2. Red box indicates the region of the original blot used in main figure.

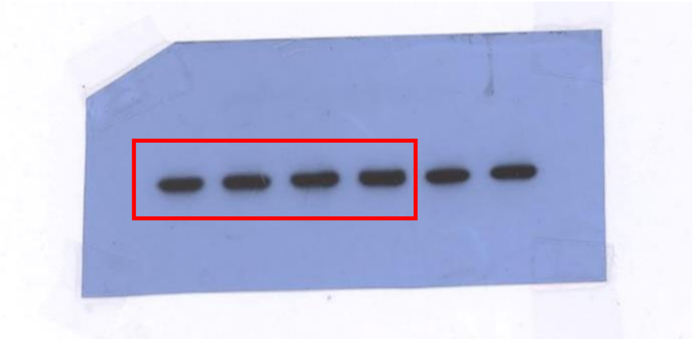

**OXY (μM)**	-	-	10	20	10	20
**Rotenone (15 μM)**	-	+	+	+	-	-

Supplementary Figure S1.12. The original full blots of β-actin for Cytochrome C. Red box indicates the region of the original blot used in main figure.
