# Supplementary material for: Untargeted metabolomics reveal pathways associated with neuroprotective effect of oxyresveratrol in SH-SY5Y cells
Source: Sci Rep. 2023 Nov 21;13:20385. doi: 10.1038/s41598-023-47558-y (PMC10663518; doi:10.1038/s41598-023-47558-y)
Supplement: Supplementary file 2 — Supplementary Figure S2. [file 41598_2023_47558_MOESM2_ESM.pdf]

Untargeted metabolomics reveal pathways associated with neuroprotective effect of oxyresveratrol in SH-SY5Y cells

Nureesun Mahamud[1,2], Phanit Songvut[3], Chawanphat Muangnoi[4], Ratchanee Rodsiri[5,6], Winai Dahlan[2] & Rossarin Tansawat[1,7]*

[1] Department of Food and Pharmaceutical Chemistry, Faculty of Pharmaceutical Sciences, Chulalongkorn University, Bangkok, 10330, Thailand.
[2] The Halal Science Center, Chulalongkorn University, Bangkok, 10330, Thailand.
[3] Laboratory of Pharmacology, Chulabhorn Research Institute, Bangkok, 10210, Thailand.
[4] Cell and Animal Model Unit, Institute of Nutrition, Mahidol University, Nakhon Pathom, 73170, Thailand.
[5] Department of Pharmacology and Physiology, Faculty of Pharmaceutical Sciences, Chulalongkorn University, Bangkok, 10330, Thailand.
[6] Preclinical Toxicity and Efficacy, Assessment of Medicines and Chemicals Research Unit, Chulalongkorn University, Bangkok, 10330, Thailand
[7] Metabolomics for Life Sciences Research Unit, Chulalongkorn University, Bangkok, 10330, Thailand

* Corresponding authors: rossarin.t@Pharm.Chula.ac.th

Corresponding authors:
Rossarin Tansawat, PhD
Department of Food and Pharmaceutical Chemistry
Faculty of Pharmaceutical Sciences
Chulalongkorn University
254 Phayathai Road, Wangmai, Pathumwan
Bangkok 10330 Thailand
rossarin.t@pharm.chula.ac.th

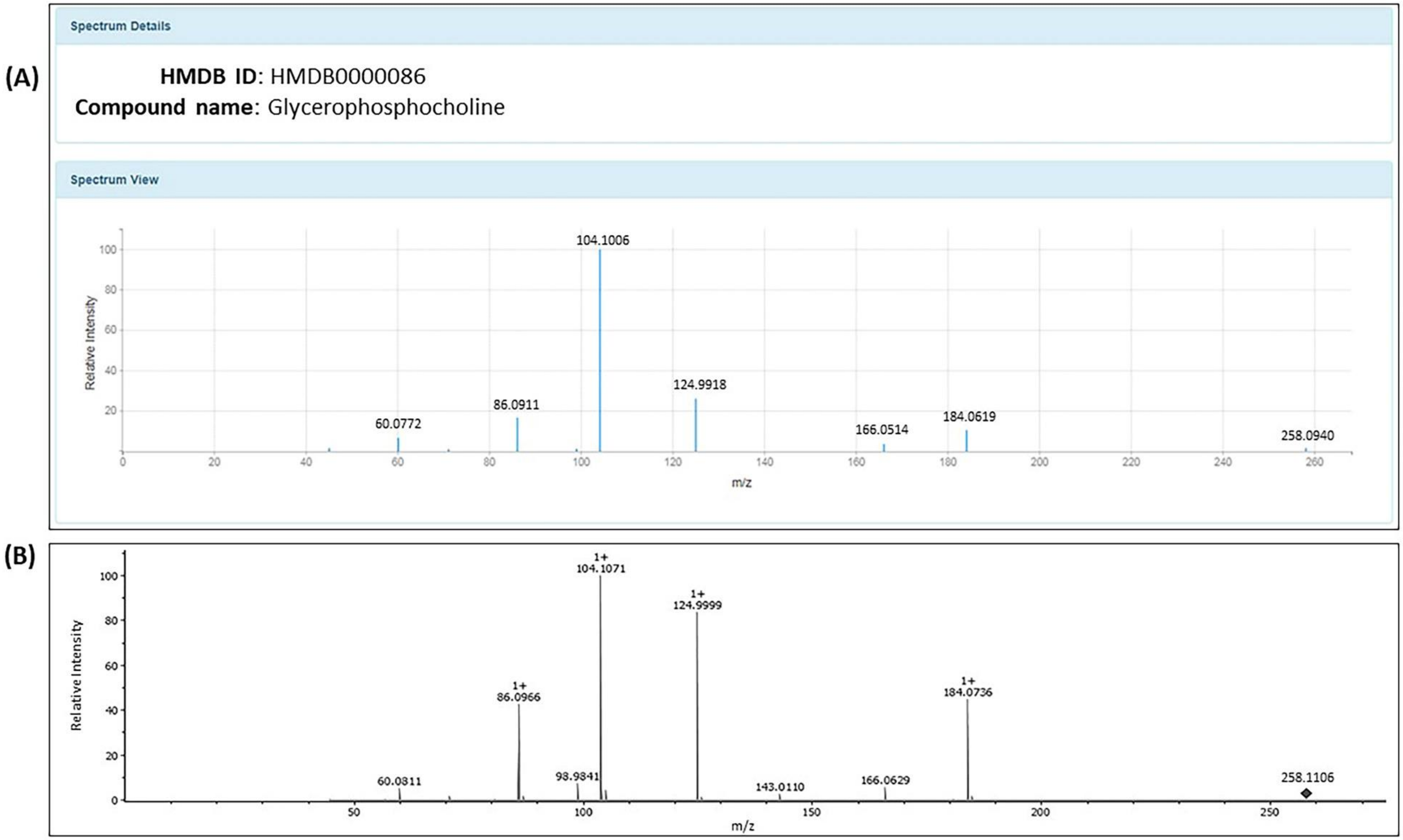

Supplementary Figure S2.1. Secondary mass spectra of glycerophosphocholine from Human Metabolome Database (HMDB) (A), from LC-QTOF-MS/MS in this study (B).

(C)

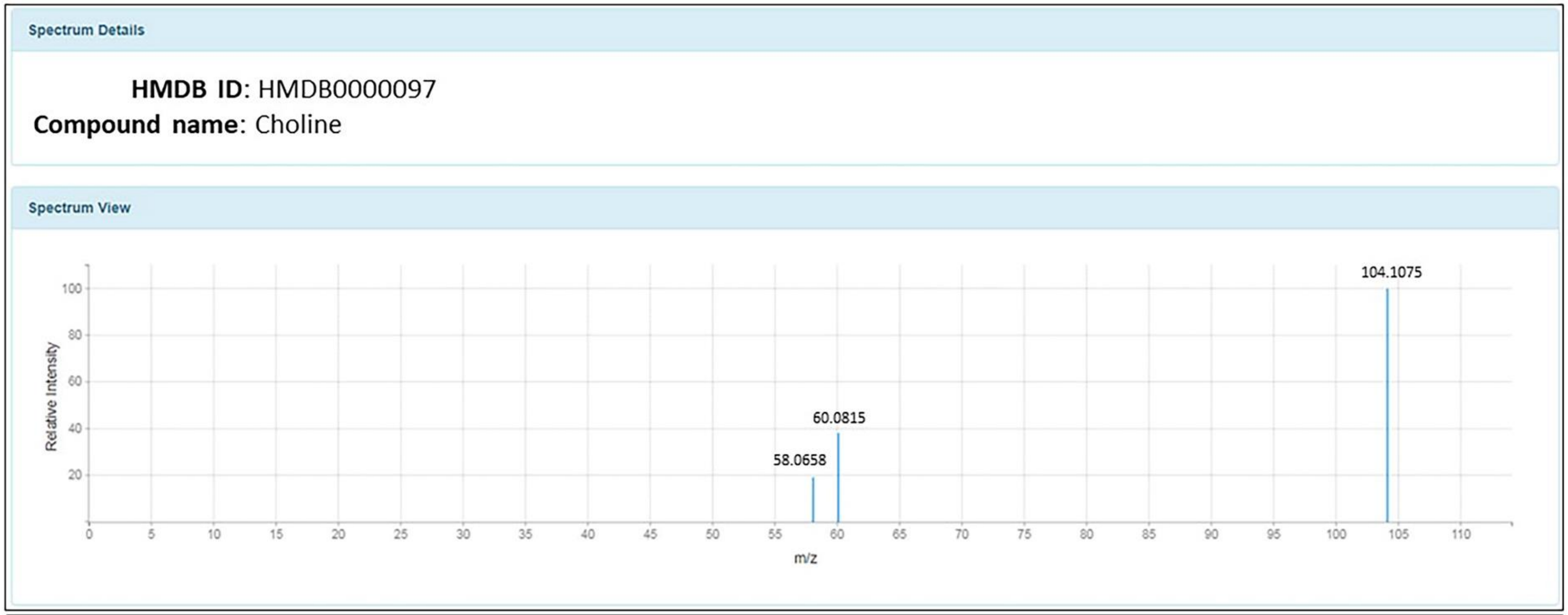

(D)

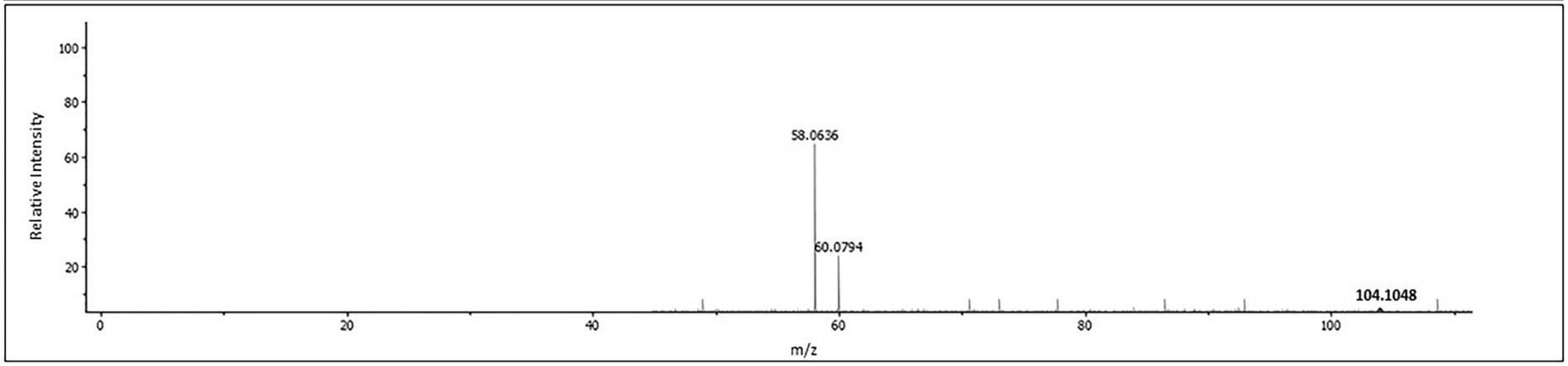

Supplementary Figure S2.2. Secondary mass spectra of choline from Human Metabolome Database (HMDB) (C), from LC-QTOF-MS/MS in this study (D).
