# Supplementary material for: Untargeted metabolomics reveal pathways associated with neuroprotective effect of oxyresveratrol in SH-SY5Y cells
Source: Sci Rep. 2023 Nov 21;13:20385. doi: 10.1038/s41598-023-47558-y (PMC10663518; doi:10.1038/s41598-023-47558-y)
Supplement: Supplementary file 4 — Supplementary Figure S4. [file 41598_2023_47558_MOESM4_ESM.pdf]

Untargeted metabolomics reveal pathways associated with neuroprotective effect of oxyresveratrol in SH-SY5Y cells

Nureesun Mahamud[1,2], Phanit Songvut[3], Chawanphat Muangnoi[4], Ratchanee Rodsiri[5,6], Winai Dahlan[2] & Rossarin Tansawat[1,7]*

[1] Department of Food and Pharmaceutical Chemistry, Faculty of Pharmaceutical Sciences, Chulalongkorn University, Bangkok, 10330, Thailand.
[2] The Halal Science Center, Chulalongkorn University, Bangkok, 10330, Thailand.
[3] Laboratory of Pharmacology, Chulabhorn Research Institute, Bangkok, 10210, Thailand.
[4] Cell and Animal Model Unit, Institute of Nutrition, Mahidol University, Nakhon Pathom, 73170, Thailand.
[5] Department of Pharmacology and Physiology, Faculty of Pharmaceutical Sciences, Chulalongkorn University, Bangkok, 10330, Thailand.
[6] Preclinical Toxicity and Efficacy, Assessment of Medicines and Chemicals Research Unit, Chulalongkorn University, Bangkok, 10330, Thailand
[7] Metabolomics for Life Sciences Research Unit, Chulalongkorn University, Bangkok, 10330, Thailand

* Corresponding author: rossarin.t@Pharm.Chula.ac.th

Corresponding author:
Rossarin Tansawat, PhD
Department of Food and Pharmaceutical Chemistry
Faculty of Pharmaceutical Sciences
Chulalongkorn University
254 Phayathai Road, Wangmai, Pathumwan
Bangkok 10330 Thailand
rossarin.t@pharm.chula.ac.th

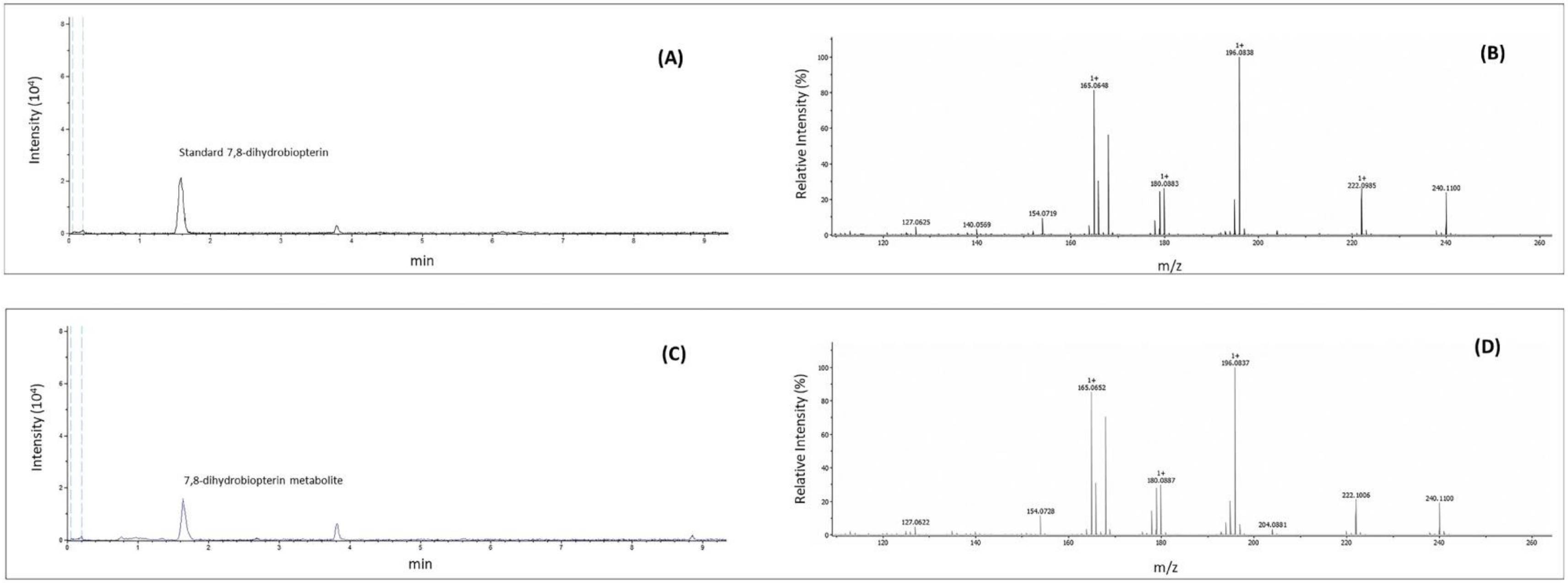

Supplementary Figure S4. The standard compound of 7,8-dihydrobiopterin from sigma (CAS number: 6779-87-9), chemical formula is $C_9H_{13}N_5O_3$. (A) Extracted ion chromatogram (EIC) of the standard compound of 7,8-dihydrobiopterin at 1 ng/µL. (B) Secondary mass spectra of the standard compound of 7,8-dihydrobiopterin at a concentration of 1 ng/µL. (C) Extracted ion chromatogram of 7,8-dihydrobiopterin from the extracted intracellular metabolite. (D) Secondary mass spectra of 7,8-dihydrobiopterin from the extracted intracellular metabolite.
