# Supplementary material for: Untargeted metabolomics reveal pathways associated with neuroprotective effect of oxyresveratrol in SH-SY5Y cells
Source: Sci Rep. 2023 Nov 21;13:20385. doi: 10.1038/s41598-023-47558-y (PMC10663518; doi:10.1038/s41598-023-47558-y)
Supplement: Supplementary file 6 — Supplementary Table S2. [file 41598_2023_47558_MOESM6_ESM.pdf]

Untargeted metabolomics reveal pathways associated with neuroprotective effect of oxyresveratrol in SH-SY5Y cells

Nureesun Mahamud[1,2], Phanit Songvut[3], Chawanphat Muangnoi[4], Ratchanee Rodsiri[5,6], Winai Dahlan[2] & Rossarin Tansawat[1,7]*

[1] Department of Food and Pharmaceutical Chemistry, Faculty of Pharmaceutical Sciences, Chulalongkorn University, Bangkok, 10330, Thailand.
[2] The Halal Science Center, Chulalongkorn University, Bangkok, 10330, Thailand.
[3] Laboratory of Pharmacology, Chulabhorn Research Institute, Bangkok, 10210, Thailand.
[4] Cell and Animal Model Unit, Institute of Nutrition, Mahidol University, Nakhon Pathom, 73170, Thailand.
[5] Department of Pharmacology and Physiology, Faculty of Pharmaceutical Sciences, Chulalongkorn University, Bangkok, 10330, Thailand.
[6] Preclinical Toxicity and Efficacy, Assessment of Medicines and Chemicals Research Unit, Chulalongkorn University, Bangkok, 10330, Thailand
[7] Metabolomics for Life Sciences Research Unit, Chulalongkorn University, Bangkok, 10330, Thailand

* Corresponding author: rossarin.t@Pharm.Chula.ac.th

Corresponding author:
Rossarin Tansawat, PhD
Department of Food and Pharmaceutical Chemistry
Faculty of Pharmaceutical Sciences
Chulalongkorn University
254 Phayathai Road, Wangmai, Pathumwan
Bangkok 10330 Thailand
rossarin.t@pharm.chula.ac.th

Supplementary Table S2. List of pathways associated with neuroprotective effect of oxyresveratrol in SH-SY5Y cells (Raw $P < 0.05$).

**Pathway**	**Impact**	**Raw *p***
Phenylalanine, tyrosine and tryptophan biosynthesis	1	0.025845
Alanine, aspartate and glutamate metabolism	0.73398	5.82E-05
Nicotinate and nicotinamide metabolism	0.42895	0.0024549
Arginine biosynthesis	0.42132	0.00017954
Arginine and proline metabolism	0.35224	3.78E-06
Vitamin B6 metabolism	0.33333	0.019782
Glycine, serine and threonine metabolism	0.32225	0.02248
Tryptophan metabolism	0.25179	0.019488
Pantothenate and CoA biosynthesis	0.23928	0.0076055
Pyrimidine metabolism	0.23844	0.047309
Glutathione metabolism	0.23285	5.82E-05
Glycerophospholipid metabolism	0.22443	0.0096355
Purine metabolism	0.20049	1.65E-05
Glyoxylate and dicarboxylate metabolism	0.16138	0.019458
Aminoacyl-tRNA biosynthesis	0	1.61E-06
Valine, leucine and isoleucine biosynthesis	0	0.001212
