# Supplementary material for: Untargeted metabolomics reveal pathways associated with neuroprotective effect of oxyresveratrol in SH-SY5Y cells
Source: Sci Rep. 2023 Nov 21;13:20385. doi: 10.1038/s41598-023-47558-y (PMC10663518; doi:10.1038/s41598-023-47558-y)
Supplement: Supplementary file 7 — Supplementary Table S3. [file 41598_2023_47558_MOESM7_ESM.pdf]

Untargeted metabolomics reveal pathways associated with neuroprotective effect of oxyresveratrol in SH-SY5Y cells

Nureesun Mahamud[1,2], Phanit Songvut[3], Chawanphat Muangnoi[4], Ratchanee Rodsiri[5,6], Winai Dahlan[2] & Rossarin Tansawat[1,7]*

[1] Department of Food and Pharmaceutical Chemistry, Faculty of Pharmaceutical Sciences, Chulalongkorn University, Bangkok, 10330, Thailand.
[2] The Halal Science Center, Chulalongkorn University, Bangkok, 10330, Thailand.
[3] Laboratory of Pharmacology, Chulabhorn Research Institute, Bangkok, 10210, Thailand.
[4] Cell and Animal Model Unit, Institute of Nutrition, Mahidol University, Nakhon Pathom, 73170, Thailand.
[5] Department of Pharmacology and Physiology, Faculty of Pharmaceutical Sciences, Chulalongkorn University, Bangkok, 10330, Thailand.
[6] Preclinical Toxicity and Efficacy, Assessment of Medicines and Chemicals Research Unit, Chulalongkorn University, Bangkok, 10330, Thailand
[7] Metabolomics for Life Sciences Research Unit, Chulalongkorn University, Bangkok, 10330, Thailand

* Corresponding author: rossarin.t@Pharm.Chula.ac.th

Corresponding author:
Rossarin Tansawat, PhD
Department of Food and Pharmaceutical Chemistry
Faculty of Pharmaceutical Sciences
Chulalongkorn University
254 Phayathai Road, Wangmai, Pathumwan
Bangkok 10330 Thailand
rossarin.t@pharm.chula.ac.th

Supplementary Table S3. Tukey HSD post-hoc test for the concentration of 7,8-dihydrobiopterin metabolite using LC-QTOF-MS/MS targeted metabolomics approach.

**Treatment groups**	**Intensity** (mean ± S.D.)
Control	17,565.25 ± 548.65[a]
Rotenone	3,505.75 ± 1,254.12[c]
OXY20+R	9,097.00 ± 914.03[b]
OXY20	16,362.50 ± 2,977.71[a]

Different letters indicate significant differences at $p < 0.05$, n = 4.
Control (0.5% DMSO);
Rotenone (IC_{50});
OXY20+R (oxyresveratrol pre-treated group at 20 µM before being exposed to rotenone);
OXY20 (oxyresveratrol pre-treated group at 20 µM without being exposed to rotenone).
